# A Comparison of Tools That Identify Tumor Cells by Inferring Copy Number Variations from Single-Cell Experiments in Pancreatic Ductal Adenocarcinoma

**DOI:** 10.3390/biomedicines12081759

**Published:** 2024-08-05

**Authors:** Daisy J. A. Oketch, Matteo Giulietti, Francesco Piva

**Affiliations:** Department of Specialistic Clinical and Odontostomatological Sciences, Polytechnic University of Marche, 60131 Ancona, Italy

**Keywords:** pancreatic ductal adenocarcinoma (PDAC), single-cell RNA sequencing (scRNA-seq), copy number variations (CNVs)

## Abstract

Single-cell RNA sequencing (scRNA-seq) technique has enabled detailed analysis of gene expression at the single cell level, enhancing the understanding of subtle mechanisms that underly pathologies and drug resistance. To derive such biological meaning from sequencing data in oncology, some critical processing must be performed, including identification of the tumor cells by markers and algorithms that infer copy number variations (CNVs). We compared the performance of sciCNV, InferCNV, CopyKAT and SCEVAN tools that identify tumor cells by inferring CNVs from scRNA-seq data. Sequencing data from Pancreatic Ductal Adenocarcinoma (PDAC) patients, adjacent and healthy tissues were analyzed, and the predicted tumor cells were compared to those identified by well-assessed PDAC markers. Results from InferCNV, CopyKAT and SCEVAN overlapped by less than 30% with InferCNV showing the highest sensitivity (0.72) and SCEVAN the highest specificity (0.75). We show that the predictions are highly dependent on the sample and the software used, and that they return so many false positives hence are of little use in verifying or filtering predictions made via tumor biomarkers. We highlight how critical this processing can be, warn against the blind use of these software and point out the great need for more reliable algorithms.

## 1. Introduction

Pancreatic ductal adenocarcinoma (PDAC) is the most common neoplastic disease of the pancreas. It is classified as one of the most chemoresistant types of cancer due to its broad intra-tumoral genetic and functional heterogeneity and characteristic dense stromal environment [1,2]. Despite extensive work toward improving diagnostic techniques, surgical procedures, and chemotherapy, the prognosis of PDAC patients is still poor with a 5-year overall survival (OS) rate of ∼13% [3], indicating an urgent need for the development of effective interventions or new targeted therapies.

Bulk genomic analysis and expression profiling of clinical specimens have shaped much of our understanding of PDAC in patients, including epigenetic changes, gene mutations and copy number alterations that facilitate clonal selection and eventually contribute to the malignant phenotype of the cancer cells [4]. With the advent of single-cell RNA sequencing (scRNA-seq), it is possible to determine the transcriptome of individual cells, allowing the identification of cell populations and their functions and bringing into order the apparent chaos, which made it seem like every cell was very different from the others and therefore carried out its activity in a manner that was poorly coordinated with the other tumor cells.

In 2009, the first study using scRNA-seq was published [5], and since then, numerous studies have been conducted by specialized laboratories with expertise in wet-lab single-cell genomics and bioinformatics. Moreover, the availability of various scRNA-seq platforms and the continuous improvement of bioinformatics methods have now made it possible for any biomedical researcher or clinician to use scRNA-seq and make new discoveries [6].

In PDAC, scRNA-seq is being used for the dissection of heterogeneity in the cell populations within the tumor and promises to reveal correlations between the presence of cell populations and prognosis and guide better clinical decisions for personalized treatment. For example, scRNA-seq performed on cells of primary tissues from ten different patients with localized and metastatic PDAC [7] showed distinct cell types including tumor cells, immune cells, cancer-associated fibroblasts (CAFs), and endothelial cells. Variations in gene expression patterns among patients were detected and it was possible to distinguish two populations of tumor cells. One population displayed EMT (epithelial–mesenchymal transition) characteristics and the other exhibited epithelial characteristics. A more aggressive form of the disease and a worse prognosis was linked to the presence of the EMT tumor cell population. In addition, the expression profile of immune cells (macrophages and T cells) from the different individuals showed high similarities, in both primary and metastatic tumors. Three major CAF clusters were also identified on the basis of gene expression, supporting the existence of three functional subtypes of CAFs: antigen-presenting CAFs (apCAFs), inflammatory CAFs (iCAFs), and myofibroblastic CAFs (myCAFs) in PDAC tumors [8,9]. A high expression of the signature genes associated with one of the CAF subtypes (myCAFs) negatively correlated with patient survival [7].

It should be noted that by scRNA-seq, the transcriptomic profile of individual cells can be read, and the sequencing data subsequently processed using bioinformatic tools to identify the cell types [10]. This identification procedure is not trivial, and the results returned are not unique due to the incomplete knowledge of the transcriptomic signatures that identify a cell type and the low sequencing depth of scRNA-seq. This sequencing limitation does not reveal the expression of all genes within a cell and therefore some transcriptomic signatures may not be found, and this may in turn affect the accurate identification of cell types [11]. Currently, the identification of malignant cells from scRNA-seq data is performed by marker-based methods and by inferring Copy Number Variations (CNVs) [12,13].

Tumor marker genes refer to genes that are predominantly expressed in a malignant tissue but are expressed very lowly or not at all in normal tissues, and marker-based methods aim to identify cancer cells or clusters based on the expression of these canonical cancer-specific marker genes [14].

However, all the markers capable of identifying PDAC cells are probably not known yet and attempts to identify them are faced with various challenges. For instance, tumors are complex tissues surrounded by a heterogeneous cellular microenvironment in which the cancer cells interact [15] and therefore it is important to analyze biopsies and not cell cultures. However, the RNA-seq technique has the intrinsic limitation of returning average data of all cell types present in the sample [16]. To overcome this limitation, more advanced studies have dissected the biopsy, for example by immunohistochemical (IHC) assay, and selected the part with the highest concentration of tumor cells in an attempt to obtain more tumor-specific sequencing data [17]. 

Furthermore, correct use of the known markers [18,19] to identify tumor cells is often not known because some of these markers only characterize tumor subpopulations and therefore do not necessarily need to be present in every cell. In addition, the weight to assign to the presence of each specific marker to reach an acceptance threshold is not known. Therefore, we still cannot rely solely on markers as an accurate method of identifying tumor cells [20,21].

In PDAC, for example, the identification of tumor cells often involves the use of epithelial markers (e.g., *E-Cadherin* or *EpCAM*) as well as intracellular markers such as the lineage-specific cytokeratin subtypes. The epithelial cell adhesion molecule (EpCAM) protein is often expressed on normal epithelial cells and is overexpressed in a subset of tumor cells such as adenocarcinomas of the stomach, colon, prostate, and pancreas [22,23,24]. However, these genes may not always be highly expressed by cancer cells and hence give rise to false negatives.

Copy number variations (CNVs) are the predominant form of variation resulting from the genomic rearrangements referred to as structural variation (SV). CNVs can lead to gene amplifications (copy number gain) and deletions (copy number loss) that could correspond to increases or decreases in the amount of RNAs and proteins encoded by the gene [25]. CNVs have been described as a hallmark of all cancers, with several studies demonstrating that variations in the number of copies of specific genes (especially for oncogenes and tumor suppressor genes) play a role in tumor initiation, development, progression, drug sensitivity and resistance by affecting the expression levels of these genes [26,27,28,29]. The occurrence of CNVs in PDAC have also been studied, including those with pathogenic roles in the development and progression of the disease [30,31], and their detection leveraged to facilitate the identification of tumor cells from scRNA-seq experiments [32]. This is accomplished through the various software available for inferring CNVs from scRNA-seq data such as sciCNV [33], InferCNV, CopyKAT [13] and SCEVAN [34].

In the literature about scRNA-seq, tumor cells are identified using markers and then these results are confirmed using software that predicts the presence of CNVs. Therefore, it is assumed that the cells considered as cancerous are those identified both by biomarker methods and by the inference of CNVs.

In this work we analyzed PDAC scRNA-seq data to verify the concordance of the predictions based on biomarkers and those based on inferring CNVs, and which of the CNV inference tools provides the best performance.

## 2. Materials and Methods

### 2.1. Dataset

We downloaded a publicly available PDAC scRNA-seq dataset (Accession # GSE154778) [7] from the Gene Expression Omnibus (GEO) database. This dataset had a total of 16 PDAC samples, of which we selected only 5 samples since the SCEVAN tool could not analyze samples with <1090 cells. In addition, normal and adjacent normal pancreas samples (GEO accession GSE165399 and GSE155698) [35,36] were also downloaded and included as control non-malignant samples intended to represent normal pancreatic cells. The PDAC and AdjNorm datasets had been similarly processed, in fact the freshly harvested tissues had been mechanically and enzymatically dissociated using a kit (PDAC samples) or collagenase P (AdjNorm samples), followed by single-cell whole transcriptome profiling using the Chromium Single Cell Gene Expression Solution system by 10x Genomics. Data processing, quality control, and analysis were subsequently performed using CellRanger (10x Genomics) and the Seurat R package (v3.0). The sequencing depth for the PDAC and AdjNorm samples was 50,000 and 100,000 reads per cell, respectively. For the normal pancreas dataset (GSE165399), freshly harvested tissues were dissociated and GEXSCOPE Single-Cell RNA Library Kit was used to construct the scRNA-seq libraries. Raw reads were processed to generate gene expression profiles using an internal pipeline and the sequencing depth for this dataset has not been reported.

A total of 8 samples were downloaded, including 3 primary PDAC (from PDAC_1 to PDAC_3) tumor samples, 2 liver metastasis PDAC samples (PDAC_4 and PDAC_5), 2 adjacent normal pancreas samples (AdjNorm_1 and AdjNorm_2) and 1 normal pancreas sample (Normal_N1). In particular, the 10x Genomics files (barcodes.tsv, features.tsv and matrix.mtx) were available for the five PDAC patients and the two samples adjacent to the tumor, while for the completely healthy sample the Raw count matrix file generated using GENXSCOPE technology was available. Table 1 shows the clinical histopathological parameters of the samples we analyzed.

### 2.2. Cell Annotation

The three 10x scRNA-seq output files of each sample were merged into a single matrix via an R script (*SeuratObject*) to have the data in a format suitable for downstream processing by cell recognition programs. The cell type annotation was conducted using SingleR tool along with the Human Primary Cell Atlas built-in reference [37]. SingleR runs on the R programming language environment and performs unbiased cell type recognition by leveraging reference transcriptomic datasets of pure cell types to independently infer the cell of origin of each single cell. In particular, SingleR uses the *celledex* package to provide access to several reference datasets (mostly derived from bulk RNA-seq or microarray data) through dedicated retrieval functions [37,38].

### 2.3. Tumor Marker Analysis

We carried out a literature search in PubMed for published papers on PDAC-specific gene markers in humans since 2006. Publications that did not conduct validation studies, included less than 5 patients, solely used cell cultures, or exclusively relied on bioinformatic methods were excluded. The markers we retrieved, along with their corresponding references, are reported in Supplementary Table S1.

We then used the Trailmaker browser to highlight cell clusters in which the tumor biomarkers were present https://www.parsebiosciences.com/data-analysis/ (accessed on 1 July 2024). Trailmaker is an open source, cloud-based analytics tool for the processing, analysis, and visualization of scRNAseq data. After data upload, a default data processing pipeline was applied to the dataset, consisting of linear steps in the “Data Processing” module. The settings in each of the steps can be adjusted to tune the analysis results. The output obtained from each step within this module served as the input for the subsequent step. Some steps (Classifier filter, Cell size distribution filter, Mitochondrial content filter, Number of Genes vs. UMIs filter, and Doublet filter, respectively) primarily involved the use of filters to eliminate unwanted and low-quality data from each sample. The final steps involved dimensionality reduction and embedding configuration (e.g., t-SNE, UMAP) followed by clustering. The “Louvain clusters” parameter was adjusted for each of the samples by using the “Data Exploration” module of Trailmaker to assess and obtain the minimum number of clusters with the most different expression profiles. We then analyzed and visualized the expression of the PDAC marker-genes in our datasets using the “Plots and Tables” modules. We considered the cell clusters expressing at least 40% of the PDAC-tumor markers as cancerous.

### 2.4. Workflows of Copy Number Variation (CNV) Analysis

CNV profiles were inferred in scRNA-seq data from all samples using the sciCNV, InferCNV, CopyKAT and SCEVAN tools, to illustrate the different patterns of chromosome copy number variation in our samples. Only these tools can process data starting from a gene expression matrix obtained from single cell experiments. All four methods are based on the hypothesis that gene expression is related to CNV occurrence. The primary objective of these methods is to eliminate the confounding influence of natural fluctuations in gene expression levels, thereby enabling a direct correlation between excess or relative gene expression and CNVs.

#### 2.4.1. SciCNV (Single-Cell-Inferred Chromosomal CNV)

SciCNV requires designated reference cells for comparisons hence we labelled the immune cells according to SingleR, as reference cells and all the other cell types as the presumed malignant cells. sciCNV (version 0.99.73) was used with the default parameters for baseline correction (0.0), sharpness resolution (1.0) and denoising (against a threshold of 0.5 copy number).

sciCNV first performs an RTAM-normalization of the gene expression for each cell and then compares the results to that of control diploid cells to calculate expression disparity scores. In order to offset the sparsity within each cell, these scores are then averaged within a genomic moving window. The sciCNV profiles of individual cells from the control and test groups are scaled by the mean sciCNV results of the test cells to convert CNV values into integers. Subsequently, denoising is performed on the scaled CNV signals at each genomic locus. Each cell’s sciCNV profile is then standardized based on the median values of its nearest neighbor to minimize random noise in the resulting sciCNV data [33].

Although scRNA-seq has allowed the comparisons of gene expression among cells, data normalization is a crucial component that determines the accuracy of these comparisons. Given the ongoing debate surrounding the best methods for normalizing single-cell transcriptomes, sciCNV developed two approaches: RTAM-1 and RTAM-2. The RTAM normalization approach leverages the weaknesses and strengths of scRNA-seq. While lowly expressed genes may have limited resolution within single cells (because of integer transcript counts) and exhibit significant variation, highly expressed genes are more reliably detected and demonstrate finer quantization of variation in relation to intensity. RTAM uses these highly expressed genes, which have more accurate expression measurements, for the alignment of cellular transcriptomes. Genes are then ranked based on their expression in each cell, followed by standardization of the summed intensities of the top-ranked genes using gene expression adjustments that are specific to each cell, and are based either on gene expression rank (RTAM1) or gene expression intensity (RTAM2). sciCNV then leverages this RTAM normalization which improves the precision of gene expression comparisons across cells, to enhance the precision of DNA copy number detection in scRNA-seq [33].

#### 2.4.2. InferCNV

InferCNV also often requires the designation of a subset of cells as ‘normal’ cells, to be used as references for comparison. In this study, we labelled the immune cells (as widely used) predicted by SingleR as reference cells and all the other cell types as presumed malignant cells. A raw counts matrix, gene/chromosome position file and annotation file were then prepared according to data requirements https://github.com/broadinstitute/inferCNV (accessed on 6 May 2024). CNVanalysis was performed using the R package InferCNV (version 1.16.0) with the Basic Options “Cut-off” of 0.1. For downstream analysis we ran HMM to predict CNV level with the parameters; “i3 HMM” Model Type, Tumor “Subclusters” Analysis Mode, “qnorm” Tumor Subcluster Partition Method, and variable Tumor Subcluster P-values (<0.1). Using the i3 HMM Model the output CNV values from InferCNV were normalized from 0 to 2, with no CNV deviation from the reference normal cells represented by 1.

InferCNV employs a corrected moving average of gene expression data. The genes are sorted based on their absolute genomic position, initially by chromosome and then by genomic start position within the chromosome. The authors of the algorithm argue that by averaging out the expression of genes that are adjacent in the genome, the gene-specific expression variability is eliminated, resulting in profiles that accurately reflect chromosomal CNVs. For further refinement of CNV profiles of the tumor cells, the algorithm constructs a CNV profile from a well-known normal (reference) sample. For each gene and cell, the final tumor CNV profile is then obtained by subtracting the normal (reference) sample from the tumor sample [39].

#### 2.4.3. CopyKAT (Copynumber Karyotyping of Aneuploid Tumors)

The following parameters were applied in the CNV analysis using CopyKAT (v1.1.0): rawmat = exp.rawdata, id.type = “S”, ngene.chr = 5, win.size = 25, KS.cut = 0.1, sam.name = “test”, distance = “euclidean”, norm.cell.names = “”, output.seg = “FLASE”, plot.genes = “TRUE”, genome = “hg20”, and n.cores = 1.

CopyKAT uses the gene expression matrix of unique molecular identifiers (UMIs) counts as input and organizes genes according to their exact genomic positions. It then uses the raw count matrix to stabilize variance by log-Freeman Turkey Transformation (FTT) and smooth outliers by polynomial dynamic linear model (DLM), followed by hierarchical clustering to estimate which of the input cells are diploid. The identity of each cluster is determined by combining the clustering results with a predefined category of “confident normal cells”. The cluster with a significantly greater abundance of predefined normal cells is labeled as the normal (diploid) cell cluster and relative gene expression profiles are computed for the anticipated tumor cells using this normal reference. In situations where there is no notable distinction in abundance of normal cells, the algorithm resorts to a ‘GMM definition’ approach for one-by-one identification of diploid normal cells. This mode assumes a combination of three Gaussian models of gene expression in single cells to indicate genomic losses, gains, and neutral states. When the majority (at least 99%) of expressed genes are in neutral states, a cell is then classified as a “confident diploid cell”. For amplification and deletion, the output CNV values from CopyKAT are normalized between −1 and 1, respectively, with no CNV change from the reference normal cells indicated as 0 [13].

#### 2.4.4. SCEVAN (Single CELL Variational ANeuploidy Analysis)

CNV inference using SCEVAN (v1.0.1) was carried out with the following parameters: count_mtx_, par_cores = 4, SUBCLONES = TRUE, ClonalCN = TRUE, beta_vega = 1.5, and plotTree = TRUE.

SCEVAN identifies a group of highly confident, non-malignant cells and uses it to establish a copy number baseline as well as to compute a copy number alteration (CNA) matrix. The input matrix is first smoothed and segmented using a joint segmentation algorithm to obtain a series of breakpoints as well as the average gene expression between these consecutive breakpoints in each cell. An intermediate CNA matrix with dimensions m (number of cells) × n (number of genes) is then computed by replacing each expression value with the average gene expression between the consecutive breakpoints in each cell. This matrix is then subjected to hierarchical clustering to form two distinct groups. If confident normal cells are detected, all the cells within the cluster containing the highest number of such cells are classified as non-malignant. The final CNA matrix is obtained by subtracting the mean value vector of all the identified normal cells. Upon completion, the generated data frame provides detailed information for each cell including the cell’s classification as either tumor or normal, whether it is designated as a normal confident cell, and its membership within a specific clonal subpopulation [34].

In Table 2, we give an overview of the four software we used to infer CNVs from PDAC scRNA-seq expression data.

### 2.5. Technical Considerations

Labelled normal cell samples (reference cells) are not necessary for InferCNV to function; however, if they are absent, it uses the average copy number profile of all cells. Labelled or unlabeled cells can be used as references by CopyKAT. In cases where normal cells are presumed to exist but are not labelled explicitly, CopyKAT will try to infer which cells are normal by hierarchical clustering. Labelled normal cell samples are necessary for sciCNV to function while SCEVAN does not require the input of reference cells but rather identifies a group of highly confident, normal cells and uses it to establish a copy number baseline.

CopyKAT and SCEVAN primarily rely on the assumption that the presence of aneuploidy can serve to identify cancer cells. The authors of CopyKAT and SCEVAN acknowledge that this is a limitation that can result in potential misclassification if the data has only a few normal cells, or if the tumor cells are nearly diploid or harbor a minimal number of genomic alterations such as pediatric cancers and liquid cancers (e.g., leukemia) [13,34].

For CopyKat, InferCNV and sciCNV, we tried different parameters, but the results were similar and therefore we used the default parameters as recommended by the tool developers. For SCEVAN we used the beta_vega 1.5 since it returned subclones and barcodes of the predicted cancer cells for all samples thus allowing as to perform homogeneous comparisons. For example, the default beta_vega 0.5 only worked with some samples.

## 3. Results

### 3.1. Cell Composition of Primary and Metastatic Tumor Tissues and Normal Pancreas Controls

To select immune cells to use as reference cells for InferCNV and sciCNV analyses, we annotated scRNA-seq data from PDAC patients (*n* = 5), adjacent normal pancreas (*n* = 2) and normal (*n* = 1) using SingleR tool. Various cell types were identified in each sample (Table 3) including non-immune cells (e.g., epithelial cells, endothelial cells, fibroblasts, and tissue stem cells) and immune cells (e.g., macrophages, monocytes, T cells, NK cells, dendritic cells (DC), and B cells). A more detailed annotation of the cell types in each sample using SingleR is reported in Supplementary Table S2.

### 3.2. PDAC Tumor Cell Identification Using a Marker-Based Method

To predict tumor cells based on the expression of PDAC tumor markers, we collected markers genes from an online literature search which resulted in the inclusion of a total of 21 published articles (Supplementary Table S1) [7,40,41,42,43,44,45,46,47,48,49,50,51,52,53,54,55,56,57,58,59]. The studies we retrieved had been performed on biopsies from treatment-naïve PDAC patients with enrichment procedures being reported in only two of the studies. Validation studies on cell lines and datasets from The Cancer Genome Atlas (TCGA), Genotype-Tissue Expression Project (GTEx) and Gene Expression Omnibus (GEO)-NCBI databases were recorded in most of the articles. The main techniques reported in the studies were microarray, RNA-sequencing and scRNA-seq, with validations using Quantitative real-time PCR (qRT-PCR) and Immunohistochemistry (IHC). We report 134 markers, some of which are more recently discovered (e.g., *SYT8*, *VSIG2*). Only two transcriptomic signatures reported were composed of co-expressed genes: the cancer stem cell markers *CD44*, *CD24*, and *ESA*; and *TSPAN8* and *SOX9*. We do not exclude the possibility that there may be other relationships of co-presence or mutual exclusion among biomarkers to identify a cancer cell, but this information was not reported in the studies we considered.

After exclusion of the markers that were not expressed in any of our samples, we obtained a total of 91 marker genes reported to be highly enriched in PDAC. By the Trailmaker browser, we assessed the expression of these marker genes in the cell clusters of each sample in our study and pooled the cells from all the clusters predicted to be cancerous in each sample. As expected, no clusters with a tumor profile were found in the Normal_N1 sample taken from the healthy patient. However, clusters with tumor transcriptomic profiles were observed in the normal samples adjacent to the PDAC tissue (Supplementary Figure S1a–h).

However, it is known that one common source of normal control samples in cancer research is the normal adjacent tissue (NAT) [60]. Although NATs should not contain malignant tumor cells, they are frequently not histologically normal and exhibit significant inflammation and other alterations that could lead to their expression of tumor-markers [61]. For instance, some researchers analyzed the transcriptomes of tumor and NAT tissues across eight tumor types and healthy tissue of the same kind and observed that the NATs exhibited a distinct state that segregates as an intermediate between tumor and healthy tissues, possibly as a result of their inflammatory reaction to the tumor tissue [60].

### 3.3. PDAC Tumor Cell Identification Using Inference of CNVs

CNV profiles of the tumor and control samples were obtained across the four CNV inference tools. InferCNV and sciCNV do not directly identify malignant cells, but rather detect alterations in chromosomal copy numbers that assumed to be indicative of malignancy. This process requires the designation of a subset of cells as ‘normal’ cells by the user, which will be used as references for comparison and computation of the cell CNV scores. On the other hand, CopyKAT and SCEVAN use gene expression data to look for the presence of aneuploidy. The rationale behind their prediction of tumor/normal cell states is that aneuploidy is a common occurrence in human cancers (90%). Immune cells and stromal normal cells usually have diploid (2N) or nearly diploid copy number profiles, whereas cells exhibiting widespread copy number aberrations throughout the genome (aneuploidy) are classified as tumor cells [13,34,62,63].

#### 3.3.1. SciCNV

**Figure 1:**
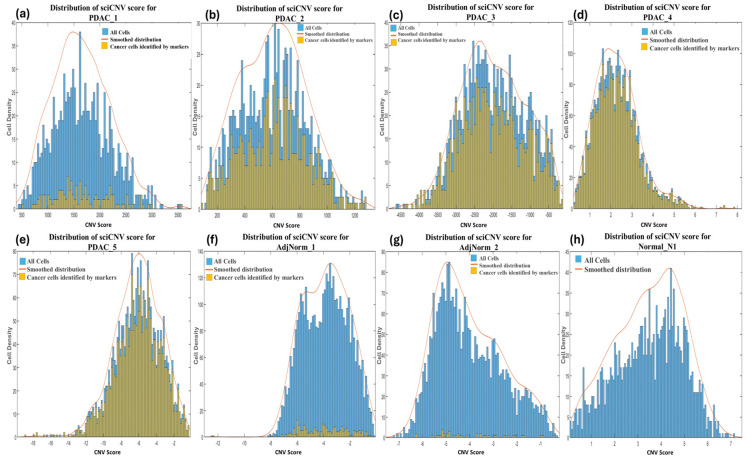
Distribution of CNV scores returned by sciCNV for each cell in primary (**a**–**c**) and metastatic (**d**,**e**) PDAC, normal adjacent tissues (**f**,**g**) and normal pancreas (**h**) samples. The histograms representing the tumor cells identified using biomarkers are shown in the color yellow, and histograms of all the cells in each sample are in blue.

SciCNV returns a score for each cell by comparing the CNV profiles of single cells to the average CNV profile of test cells. The authors of sciCNV state that the tumor CNV score enables the detection of malignant cells from normal diploid cells that harbor the CNVs specific to the tumor [33], yet no further guidance or criteria on how to accomplish this is described. We therefore retrieved the tool’s CNV score output file (TotScore) for each sample and visualized them in density plots (Figure 1a–h). However, since no separate distributions were highlighted, we were unable to distinguish the normal cells from the tumor cells based on the CNV scores. We further plotted the tumor cells predicted by markers in the sciCNV score density plots for each sample and observed that these were distributed uniformly throughout the CNV score density plots (Figure 1a–h), therefore we were unable to establish clear criteria of distinguishing the tumor cells from normal cells.

#### 3.3.2. InferCNV

**Figure 2:**
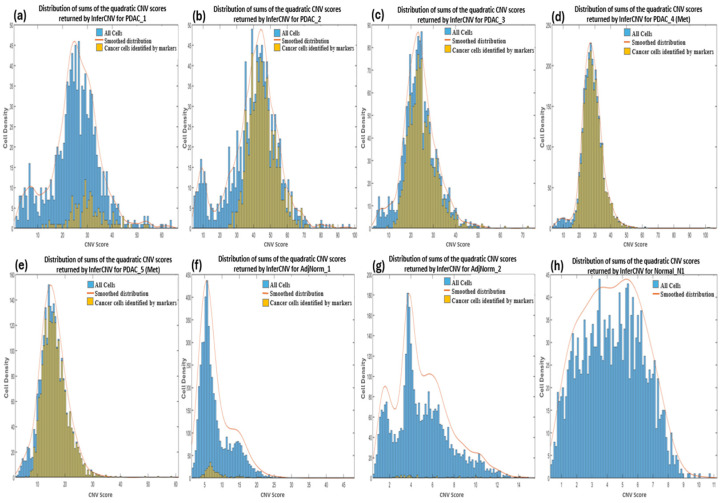
Density plots showing the distribution of sums of the quadratic CNV scores returned by InferCNV for primary (**a–c**) and metastatic (**d**,**e**) PDAC, normal adjacent (**f**,**g**) and normal pancreas (**h**) samples. The CNV score calculated as the quadratic sum of the CNV region is displayed on the x-axis.

CNV profiles of the tumor and control samples were visualized in heatmaps (Supplementary Figure S2). The amplifications observed in chromosome 6 of the reference heatmaps were expected and due to the overexpression of the major histocompatibility complex (MHC) genes characteristic of immune cells used as the reference cells in the analysis. These genes encode proteins that play a crucial role in presenting antigens derived from pathogens to the immune system of the host, and studies have suggested that they exhibit significant variation in copy numbers both within and between species [64]. We calculated the CNV score of each cell as the quadratic sum of each CNV region and visualized these as density plots (Figure 2). For most of the samples we observed a bimodal distribution which allowed us to set a threshold between the two peaks by hypothesizing that the profiles with the lowest and highest score identified normal and tumor cells, respectively. However, we also imposed that the cells to be considered as cancerous must have a CNV score greater than 12. We deduced this threshold from the observation of the CNV scores of bimodal distributions as well as of the cells derived from the normal healthy sample (Normal_N1).

#### 3.3.3. CopyKAT

Heatmaps displaying the CNV profiles across the chromosomes for each sample were generated through the CopyKAT pipeline (Supplementary Figure S3). The cells were classified as either aneuploid or diploid in the CopyKAT output and considered as cancer and normal cells, respectively, as suggested by the authors of the software.

#### 3.3.4. SCEVAN

In the SCEVAN output file, cells were defined as either normal or tumor cells, and CNV profiles of the predictions as well as subclones of the predicted tumor cells were visualized in the form of heatmaps (Supplementary Figure S4). We further noted that the frequency of amplifications exceeded that of deletions for all samples, which is in agreement with previous reports [65] and may be a result of selection on deletions [66].

### 3.4. Comparisons among the Methods of Inferring CNVs

In Table 4, we show comparisons among the number of epithelial cells, cancer cells identified by markers, their overlap, and tumor cells predicted by three CNV-based prediction tools in each sample. Here we have chosen to consider the tumor cells identified by a marker-based method as the positive reference against which to compare the results of the CNV inference algorithms.

Since it is known that PDAC cells are of the epithelial type, we expected that the number of tumor cells obtained through the markers or predicted through the increased presence of CNVs does not exceed that of epithelial cells. The reliability of these predictions was confirmed by the nearly complete overlap observed between the epithelial cells and tumor cells predicted by markers, that is, almost all predicted tumor cells are epithelial cells and majority of the epithelial cells are tumor cells.

SciCNV predictions were excluded from this comparison because it was not possible to identify a criteria or threshold (as can be observed from the distribution in Figure 1) beyond which to consider the cells as tumorous.

Regarding the predictions by CNV inference tools, InferCNV often exceeds the number of epithelial cells in PDAC patients whereas CopyKAT and SCEVAN sometimes return lower numbers suggesting a low specificity for InferCNV and a low sensibility for CopyKAT and SCEVAN.

In the normal adjacent tissue samples, assuming that cells with a transcriptomic profile that resembles that of a tumor cell are present, it can be observed that in AdjNorm_1 and AdjNorm_2, the tools predict a number of tumor cells that are more than and less than that of epithelial cells, respectively, thus highlighting a strong dependence of the predictions on the samples.

However, it is surprising that CopyKAT and SCEVAN tools indicate the presence of tumor cells in the completely normal sample (Normal_N1).

**Figure 3:**
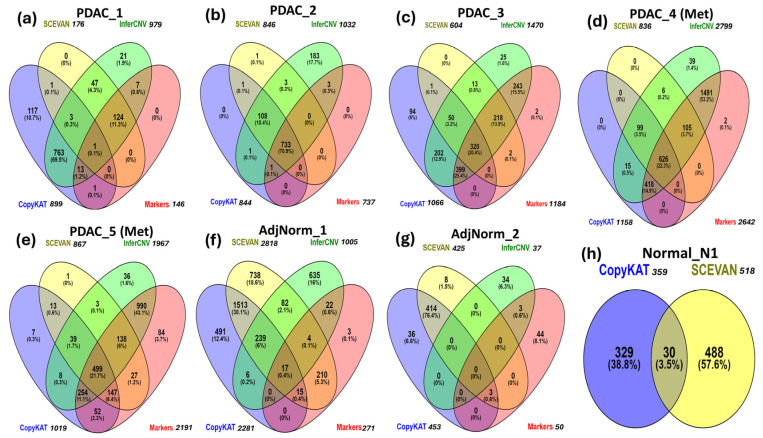
Comparison of CNV inference tools and a marker-based method for tumor-cell identification in primary (**a**–**c**) and metastatic (**d**,**e**) PDAC tumor samples, adjacent normal pancreas samples (**f**,**g**) and normal pancreas sample (**h**).

It is important to note that in Table 4, the intersections between biomarker predictions and those obtained through CNV inference tools have not been taken into consideration. For example, in the PDAC_1 patient we cannot deduce whether the 146 tumor cells identified by markers are included in the 979 cells predicted via InferCNV. This type of analysis, that is, the overlap among predictions is shown by Venn diagrams in Figure 3a–h.

In the PDAC_1 sample, most tumor cells are predicted by InferCNV and SCEVAN tools, but InferCNV and CopyKAT return the highest number of false positives. For the PDAC_2 sample, all tools detect most of the tumor cells (733, or 70.95%) and InferCNV is the software with the highest number of false positives. In PDAC_3, InferCNV and CopyKAT predict most of the tumor cells. In PDAC_4 and PDAC_5, InferCNV predicts 94.1% (2640) and 81.9% (1881) of the cancer cells, respectively. In the AdjNorm_1 and AdjNorm_2 samples, we observe the highest number of false positives predicted by CopyKAT and SCEVAN. In the normal pancreas sample Normal_N1, InferCNV does not identify any tumor cells, unlike CopyKAT and SCEVAN.

In Supplementary Tables S3–S5, we have reported data of the diagrams of Figure 3 and detailed calculations of the performance of the tools using the cells identified by markers as reference tumor cells. Performances of the three tools are summarized in Table 5. The objective was that the tumor cells predicted by the three CNV inference tools should be similar to those identified by markers, even in the AdjNorm samples.

Notably, the performances of the software are strongly sample-dependent, with InferCNV and SCEVAN being the most sensitive and the most specific tools, respectively. Since the five PDAC tumor samples were all derived from the same experiment and had the same sequencing depth, it can be assumed that the observed variations in the predictions are caused by a difference in biology of the tumors. However, almost all the software seem to lack sensitivity in the samples adjacent to the tumor tissue, but it is worth considering that these tools could have potentially identified the real tumor cells among those with an expression profile similar to that of the tumor. If so, the three prediction tools would allow us to filter the cancer cells obtained by the markers.

We performed several more analyses with different parameter settings but did not observe any significant performance improvements. However, to show how changing some of the parameters impacts the results, we report Supplementary Table S6 which shows the nonlinearity of the results as a function of parameter values.

### 3.5. Comparison between Tumor Cells Predicted by InferCNV and Markers

At this point, we wanted to verify whether the low specificity of InferCNV was due to our choice of threshold of the computed CNV score as well as whether the tumor cells identified by the markers are those with the highest CNV score, and hence confirm the assumption that CNVs are a hallmark of all cancer. InferCNV returns a score for each gene and the total scores of a cell can be calculated as the quadratic sum of the scores [67]. We show the distribution of the CNV scores of each cell in each of our samples in Figure 2a–h, distinguishing tumor cells identified by markers (yellow color) compared to the total number of cells (blue color). In the PDAC samples (Figure 2a–e), we observe all tumor cells identified by markers to be generally above the threshold (≥12) apart from PDAC_5 (Met) that present cancer cells even below it (<12). Hence the establishment of a cut-off between the two peaks in the bimodal distribution enables the complete identification of tumor cells, although this may also result in the inclusion of many normal cells. For example, the PDAC_1 sample has many normal cells (verified to be mostly immune cells) with the same CNV scores as the tumor cells therefore indicating a high number of false positives by InferCNV. The AdjNorm_1 sample also presents a bimodal distribution with the CNV score cut-off at around 12 (Figure 2f) suggesting that beyond this threshold there are tumor cells, but this assumption is contradicted by the markers. Further analysis revealed that many of these false positives were immune cells therefore suggesting that the cells predicted to be tumorous by biomarkers may be in an early stage of disease and hence have a low number of CNVs, or it may be proof that they should not be considered as tumor cells because they have fewer CNVs compared to the normal cells. As expected, cells taken from the normal pancreas tissue has a lower CNV score than those from PDAC patients (Figure 2h).

## 4. Discussion

In this work we compared different tools that analyze single cell RNA-seq data in order to facilitate the identification of tumor cells, based on the fact that CNVs are a hallmark of all cancers and therefore these cells should present a higher number of cancer-associated CNVs compared to other cells.

Tumor cells can also be identified by the presence of specific gene markers. However, even though several of these cancer biomarkers are known, there are two main challenges in their use. On one hand, certain gene markers may be patient-specific (i.e., derived from subgroups of patients). Consequently, the exclusion/inclusion relationships indicating which markers are indispensable, interchangeable or mutually exclusive to define a cancer cell remain unknown. In other words, the markers that constitute a necessary but not sufficient condition to define a tumor cell are not known.

On the other hand, the single cell RNAseq technique does not yet guarantee a good sequencing depth so some cells may not be recognized as tumor because the expression of some of their markers may not have been detected [68]. For these reasons, the authors who analyze single cell data group cells in clusters andbase the cancer cell detection not only on the expression of biomarkers but also on the presence of CNVs. To accomplish the latter, tools exist that process expression data to infer CNVs, and from these, predict which cells are tumorous. Using scRNA-seq data from primary and metastatic PDAC samples, healthy and adjacent healthy tissues, we show a poor concordance of predictions among these tools, and a strong sample-dependence. We rule out the possibility of technical differences influencing the predictions and hence only biological heterogeneity could be responsible for the observed differences. In addition, each of the algorithms we evaluated has its own strengths and weaknesses.

From our comparisons, it emerged that InferCNV algorithm has the highest sensitivity. However, significant drawbacks of InferCNV include its (i) inability to identify precise coordinates of chromosome breakpoints or copy number segments, as it can only provide smoothed averages of gene windows; (ii) manual choice and input of reference cells [69,70] which can influence the results; and (iii) long analysis time (days) even on a computer with an i9 13th gen processor.

The CopyKAT method, which classifies non-malignant and malignant cells automatically, represents a way to overcome these restrictions. Unfortunately, it showed poor sensitivity and specificity in our comparisons. This was especially evident in the PDAC_1 and Adj_Norm_1 samples in which ~80% of the predicted tumor cells were the immune cells and not epithelial cells as expected in PDAC. As already known, these problems could be because in datasets with few or no tumor cells, CopyKAT mistakenly identified CNV events in cells exhibiting the highest gene expression levels. Nevertheless, the authors of CopyKAT acknowledge this limitation and suggest that the implied CNV occurrences can be disregarded in such instances [13]**.**

SCEVAN was the fastest among all four software but is based mainly on the search of aneuploidy [34] and was unable to analyze samples with <1100 cells. SCEVAN performs a stringent filtration to work with high quality data, and its authors claim that it works well in samples with a significant amount of immune infiltration. It is therefore particularly suitable for studies that involve the analysis of heterogeneous cell populations, for example, to gain insights into the interaction between malignant cells and their microenvironment [71]. Although our data meets these recommendations, SCEVAN appears to have low sensitivity and medium specificity.

We should take into account that the identification of tumor cells based solely on inferring CNVs (without the use markers) relies on the presence of genetically unstable cells and might therefore not be able to detect all cancer cells since some of them may have nearly diploid genomes or minimal variations in their genomic structure [12,13]. As a result, most studies claim to use a combination of both marker-based methods and inference of CNVs to provide more accurate results, even though the specific criteria used remain unclear.

Nevertheless, it is important to acknowledge certain limitations in this study. First, we have a limited number of samples, but we already have a clear picture of the weakness of the predictions since high discrepancies are evident in seven out of eight samples analyzed. Second, we considered the tumor cells identified by markers as the benchmark for evaluating the results of the CNV inference software and therefore may not be completely accurate. In fact, there is lack of knowledge on how to apply tumor markers, and the problem of defining an identikit of a tumor cell is also present in pathological anatomy where many PDAC markers are available: *PTX3*, *LGALS9*, *ENO1*, *REG4*, *POSTN*, *CA242*, *LGALS1*, *SERPINB5*, *pVHL*, *CA125*, *MUC5AC*, *THBS2*, *LTBP2*, *CPA4*, *IMP3*, *CD13*, *DKK1*, *KOC*, *S100P*, *MSLN*, *MUC1*, *MUC4*, *PAM4*, *CA19-9*, *GPC-1*, *ANXA10*, *CLDN18*, *KRT19*, *KRT7*, *KRT17*, *CEA*, and *CLDN4* [72,73,74]. Here too the correct use of markers is debatable because each of the protein markers is characterized by its own sensitivity and specificity, and some markers (such as *CA19-9*) have recently proven to be less specific than we thought [74]. Third, the false positives and negatives that we have highlighted in some cases could be solely due to the parameters used in processing, but the users of these tools are not guided in choosing among the many possible parameter combinations, each of which provide different results. In addition, these settings should also take into account the total number of cells in a sample. Fourth, some tools, such as InferCNV and sciCNV, do not directly show which cells are cancerous but rather return the number of CNVs without indicating a threshold beyond which to consider a cell as tumorous. In these cases, one can hope to identify a bimodal distribution of CNV scores and establish a threshold that distinguishes the two cell populations. Fifth, although there is a formula suggested by the authors of InferCNV to process the CNV scores of each cell from InferCNV output, different formulae could be evaluated and used to increase the accuracy of tumor cell identification. Furthermore, formulas that associate a greater weight with the genes involved in cancer onset, development and progression should be evaluated. Lastly, the users of the software are not guided in choice of the best approach for accurate identification of tumor cells, that is, whether to intersect the predictions of biomarkers with those of CNVs or to use the latter to expand those found with markers alone. In other words, whether CNVs have to be used to increase the specificity of biomarker predictions or increase their sensitivity.

In conclusion, the accurate and sensitive detection of CNVs in scRNA-seq data remains challenging despite the many available algorithms, and therefore the development of more accurate and well documented tools is still needed. Programs that integrate the prediction of CNVs with the presence of mutations would also be of importance because the amplification of an oncogene has greater effect than the amplification of a proto-oncogene. Similarly, the loss of one copy of a tumor suppressor gene is much more serious if the remaining copy is mutated so it is no longer functional (the two-hit hypothesis). We would also like to encourage bioinformatics tool developers to provide better user guides and parameter explanations such as pointers on how the user should set parameters based on sample characteristics (e.g., cell count and sequencing depth).

## Figures and Tables

**Table 1:** Clinical histopathological parameters of the samples.

Patient ID	GEO ID	Age	Gender	Diagnosis	Primary or Metastasis	Stage	Grade
PDAC_1	GSM4679538	56	F	PDAC	Primary	IIA	3
PDAC_2	GSM4679539	67	F	PDAC	Primary	IIA	2
PDAC_3	GSM4679541	74	F	PDAC	Primary	IIA	2
PDAC_4	GSM4679546	46	M	PDAC	Liver Met	IV	ND
PDAC_5	GSM4679547	67	M	PDAC	Liver Met	IV	ND
AdjNorm_1	GSM4710706	–	–	Adjacent normal pancreas		–	–
AdjNorm_2	GSM4710707	–	–	Adjacent normal pancreas		–	–
Normal_N1	GSM5032773	50	M	Normal Pancreas Sample		–	–

Met: metastasis; ND: Not determined.

**Table 2:** Overview of the four methods compared for inferring CNVs from scRNA-seq data.

Method	Model	Input	Output	Tool
sciCNV	-Smoothed averages of gene windows -RTAM normalization	- UMI count - Cell annotation	- Matrix containing the inferred CNVs along with their score for each cell of the sample	R package https://rdrr.io/github/alimahdipour/sciCNV/f/vignettes/Introduction.Rmd (accessed on 6 May 2024)
InferCNV	-Smoothed averages of gene windows	- UMI count - Cell annotation - A gene/chromosome positions file	- Matrix containing the inferred CNVs for each cell of the sample	R package https://github.com/broadinstitute/inferCNV/wiki (accessed on 6 May 2024)
CopyKAT	Bayesian segmentation approach	- UMI count - Cell annotation	- Matrix containing the inferred CNVs for each cell of the sample - Cells inferred as aneuploid are considered tumoral - Cluster of the predicted cancer cells	R package https://github.com/navinlabcode/copykat (accessed on 6 May 2024)
SCEVAN	-Multichannel segmentation approach	- UMI count - Cell annotation	- Matrix containing the inferred CNVs for each cell of the sample - Tumor/normal cell classification likely based on aneuploidies - Cluster of the predicted cancer cells	R package https://github.com/AntonioDeFalco/SCEVAN (accessed on 6 May 2024)

**Table 3:** Cell type annotation for each sample using SingleR.

Cell Type	PDAC_1	PDAC_2	PDAC_3	PDAC_4 (Met.)	PDAC_5 (Met.)	AdjNorm_1	AdjNorm_2	Normal_N1
NON-IMMUNE CELLS	(34.8%)	(90%)	(93.2%)	(95.6%)	(93.0%)	(43.5%)	(43.8%)	(57.4%)
Epithelial cells	196	844	1325	2774	2303	705	653	388
Endothelial cells	10	12		1	1	156	111	170
Fibroblasts	95	83	84		1	31	8	84
Tissue stem cells	23	27	9			529	62	127
Others	58	59	45	3	6	778	782	296
IMMUNE CELLS	716 (65.2%)	114 (10.0%)	107 (6.8%)	127 (4.4%)	173 (7.0%)	2770 (55.7%)	1974 (55.0%)	790 (42.6%)
Total Number of Cells	1098	1139	1570	2905	2484	4969	3590	1855

**Table 4:** The number of epithelial cells in each sample versus the tumor cells predicted by different tools.

Sample	Total no. of Cells in Sample	Epithelial Cells (SingleR)	Cancer Cells According to Markers	Intersection between the Epithelial Cells (SingleR) and Cancer Cells Predicted by Markers *	InferCNV	CopyKAT	SCEVAN
PDAC_1	1098	196 (17.8%)	146 (13.3%)	145 (99.3%)	979 (89.1%)	899 (81.9%)	176 (16.0%)
PDAC_2	1139	844 (74.1%)	737 (64.8%)	735 (99.7%)	1032 (90.6%)	844 (74.1%)	846 (74.3%)
PDAC_3	1570	1325 (84.4%)	1184 (75.4%)	1176 (99.3%)	1470 (93.6%)	1066 (67.9%)	605 (38.5%)
PDAC_4 (Met)	2905	2774 (95.5%)	2642 (91.0%)	2639 (99.9%)	2799 (96.4%)	1158 (39.9%)	836 (28.8%)
PDAC_5 (Met)	2484	2303 (92.7%)	2191 (88.2%)	2189 (99.9%)	1967 (79.2%)	1019 (41.0%)	867 (34.9%)
AdjNorm_1	4969	705 (14.2%)	271 (5.5%)	233 (86%)	1005 (25.9%)	2282 (45.9%)	2818 (56.7%)
AdjNorm_2	3590	653 (18.2%)	50 (1.4%)	39 (78%)	37 (1.0%)	453 (12.6%)	425 (11.8%)
Normal _N1	1855	388 (20.9%)	0 (0%)	0 (0%)	0 (0%)	359 (19.4%)	518 (27.9%)

* The percentages in this column refer to the number of predicted tumor cells which are also epithelial.

**Table 5:** Performance of the CNV-inference tools using a marker-based method as the benchmark.

Sample	InferCNV		CopyKAT		SCEVAN	
	Sensitivity	Specificity	Sensitivity	Specificity	Sensitivity	Specificity
PDAC_1	0.99	0.12	0.10	0.07	0.86	0.95
PDAC_2	1.00	0.27	1.00	0.73	0.99	0.72
PDAC_3	1.00	0.25	0.61	0.10	0.46	0.83
PDAC_4 (Met)	1.00	0.40	0.40	0.57	0.28	0.60
PDAC_5 (Met)	0.86	0.71	0.43	0.77	0.37	0.81
AdjNorm_1	0.16	0.80	0.12	0.52	0.91	0.45
AdjNorm_2	0.06	0.99	0.06	0.87	0.06	0.88
Normal_N1		1.00		0.81		0.72
**MEAN**	0.72	0.57	0.39	0.55	0.56	0.75

## Data Availability

Data are contained within the article or Supplementary Material.

## Supplementary Materials

The following supporting information can be downloaded at: https://www.mdpi.com/article/10.3390/biomedicines12081759/s1. Figure S1: The identified PDAC tumor cell clusters using marker-based methods for each sample, Figure S2: InferCNV’s heatmap visualization of the CNV profiles of all samples, Figure S3: CopyKAT’s heatmap visualization of the CNV profiles of all samples, Figure S4: SCEVAN’s heatmap visualization of the predictions and subclones of the predicted tumor cells in all samples; Table S1: Pancreatic Ductal Adenocarcinoma cell markers from literature search, Table S2: A detailed cell type annotation for each sample using SingleR, Table S3: Detailed calculations of the performance of InferCNV, Table S4: Detailed calculations of the performance of CopyKAT, Table S5: Detailed calculations of the performance of SCEVAN, Table S6: The effects of variations of parameter settings on predictions.
