# Supplementary material for: A Comparison of Tools That Identify Tumor Cells by Inferring Copy Number Variations from Single-Cell Experiments in Pancreatic Ductal Adenocarcinoma
Source: Biomedicines. 2024 Aug 5;12(8):1759. doi: 10.3390/biomedicines12081759 (PMC11351975; doi:10.3390/biomedicines12081759)
Supplement: Supplementary file 1 [file biomedicines-12-01759-s001.zip › Supplementary figures and tables.pdf]

Supplementary Figure S1: The identified PDAC tumor cell clusters using marker-based methods for each sample **(a)** Clusters 2 and 6 in PDAC_1 **(b)** Clusters 0, 1, 3, 5 and 6 in PDAC_2 **(c)** Clusters 0, 1 and 3 in PDAC_3 **(d)** Clusters 0, 1, 2, 3 and 4 in PDAC_4 (Met) **(e)** Clusters 0, 1, 2, 3 and 5 in PDAC_5 (Met) **(f)** Clusters 4 in AdjNorm_1 **(g)** Cluster 10 in AdjNorm_2 **(h)** No clusters in Normal_N1.

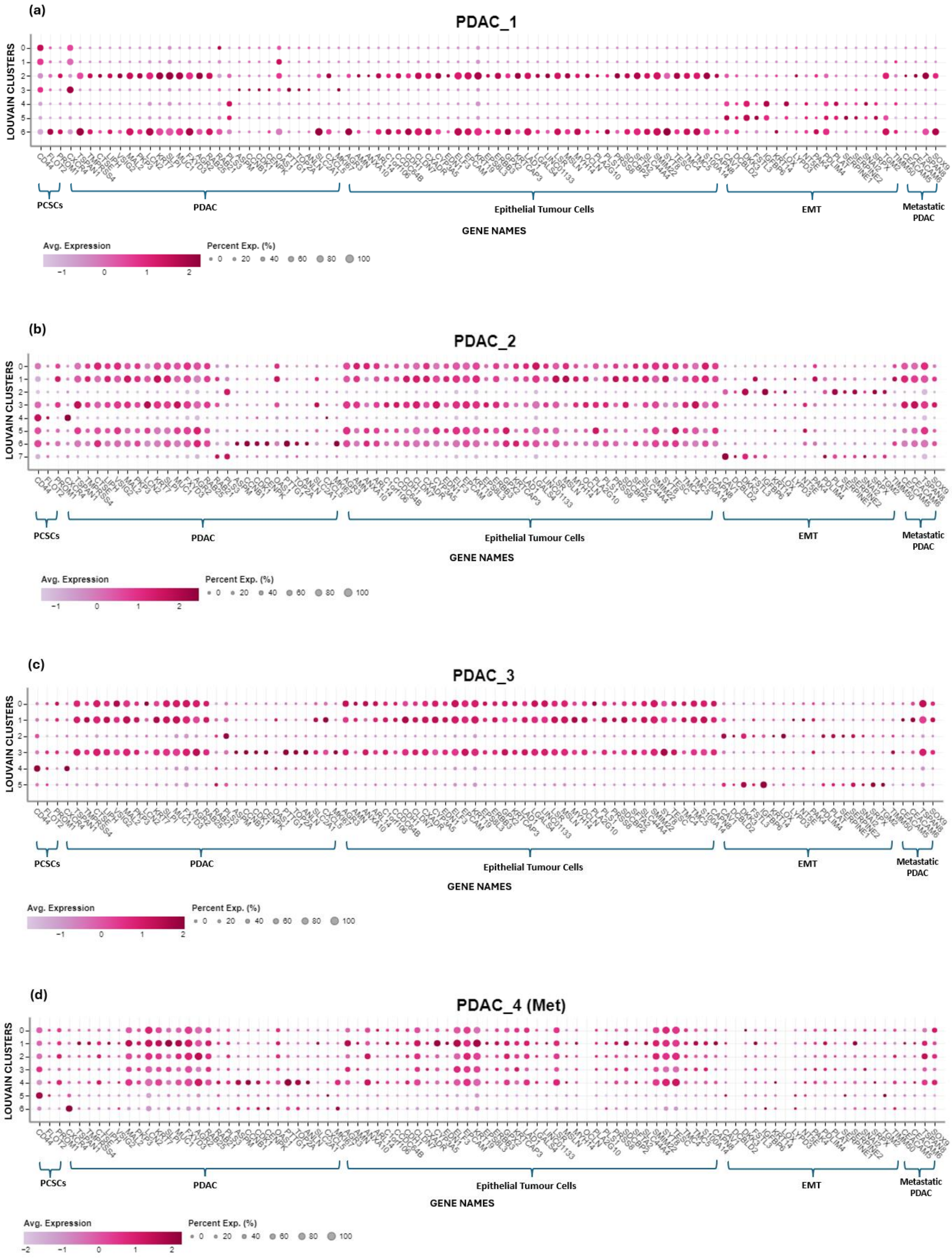

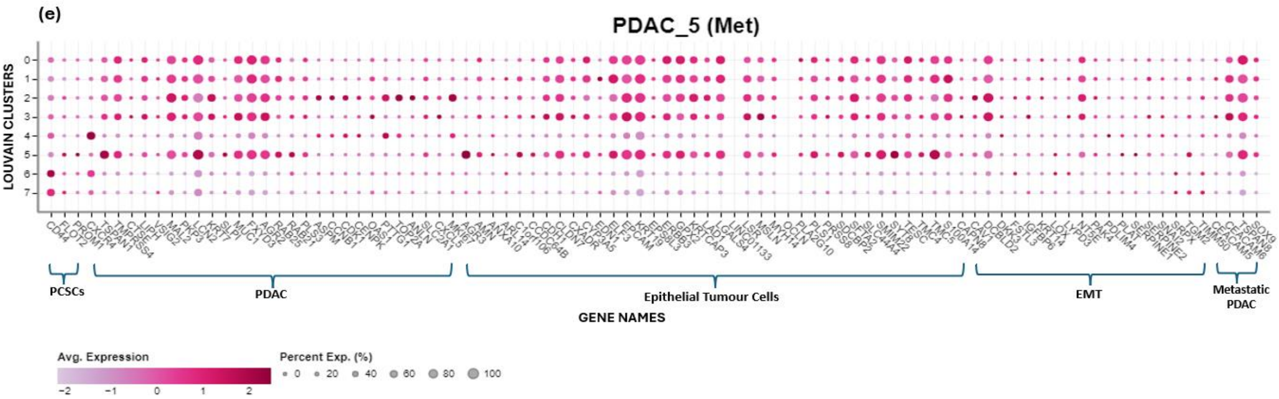

(e)
PDAC_5 (Met)
LOUVAIN CLUSTERS
PCSCs
PDAC
Epithelial Tumour Cells
EMT
Metastatic PDAC
GENE NAMES
Avg. Expression
Percent Exp. (%)

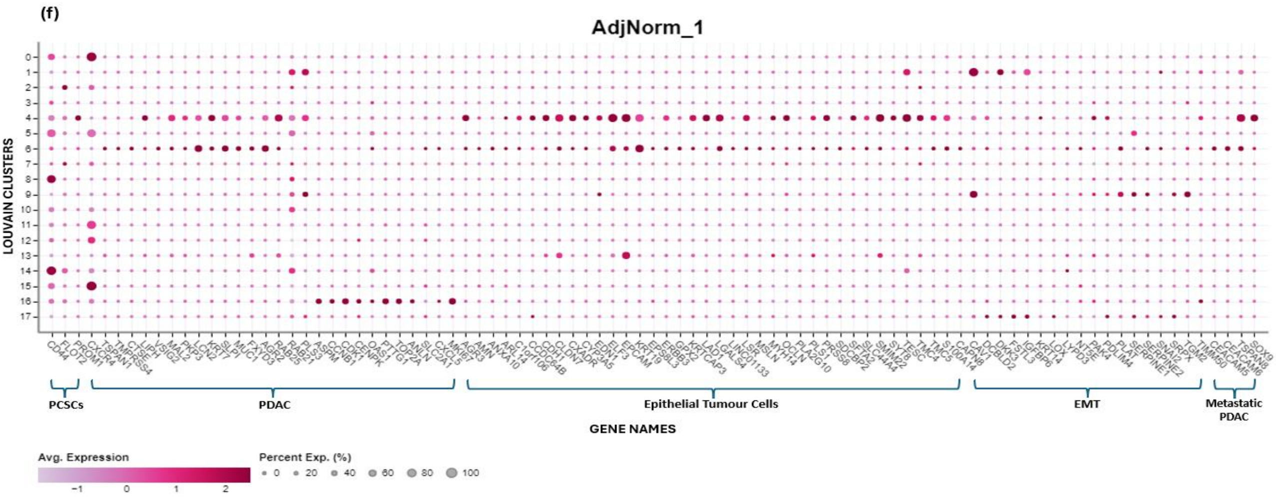

(f)
AdjNorm_1
LOUVAIN CLUSTERS
PCSCs
PDAC
Epithelial Tumour Cells
EMT
Metastatic PDAC
GENE NAMES
Avg. Expression
Percent Exp. (%)

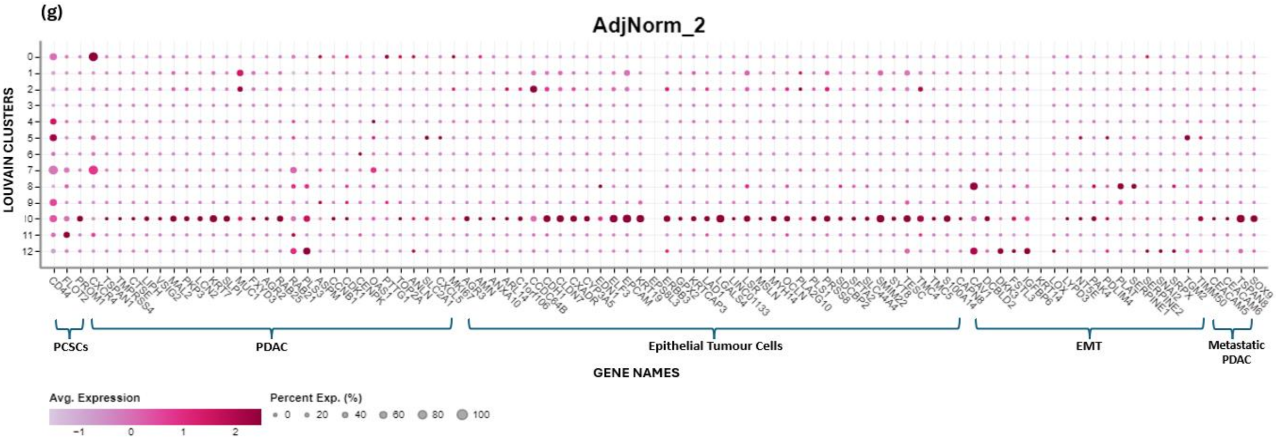

(g)
AdjNorm_2
LOUVAIN CLUSTERS
PCSCs
PDAC
Epithelial Tumour Cells
EMT
Metastatic PDAC
GENE NAMES
Avg. Expression
Percent Exp. (%)

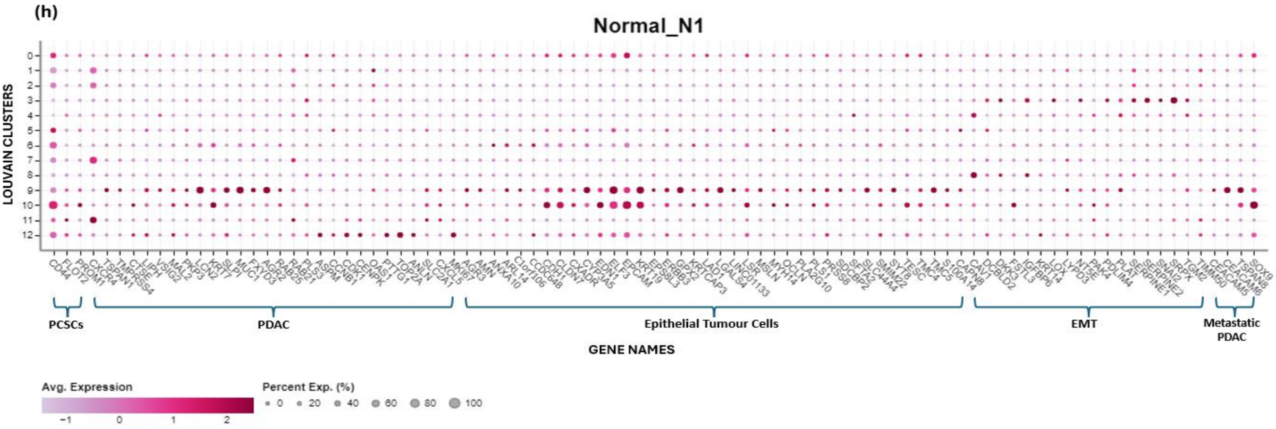

(h)
Normal_N1
LOUVAIN CLUSTERS
PCSCs
PDAC
Epithelial Tumour Cells
EMT
Metastatic PDAC
GENE NAMES
Avg. Expression
Percent Exp. (%)

Supplementary Figure S2: InferCNV's heatmap visualization of the CNV profiles of the primary PDAC tumor samples (**a-c**), metastatic PDAC tumor samples (**d-e**), and the control samples (**f-h**). The chromosomes are indicated on the x-axis, while the distinct clusters are represented on the y-axis. The top heatmaps represents the immune cells within the dataset, which were selected as the reference cells. The bottom heatmaps correspond to all the other cell types including the malignant cells, indicating the presence of distinct clonal populations characterized by shared or distinct CNVs among the different clusters. Chromosome regions displaying red color (indicating high expression) are likely amplified in these malignant cells compared to normal cells, whereas those with blue color (indicating low expression) are lost. In contrast, the "normal" cells do not show these significant variations as their genome is still diploid although with minimum structural changes.

(a)

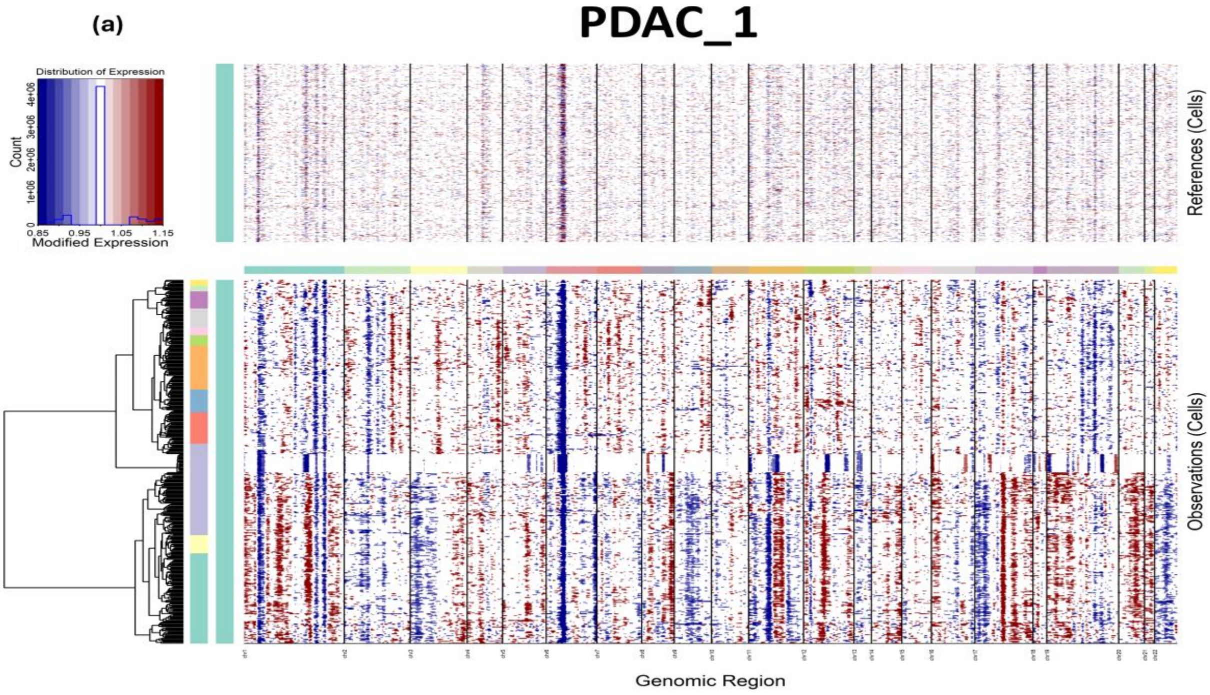

(b)

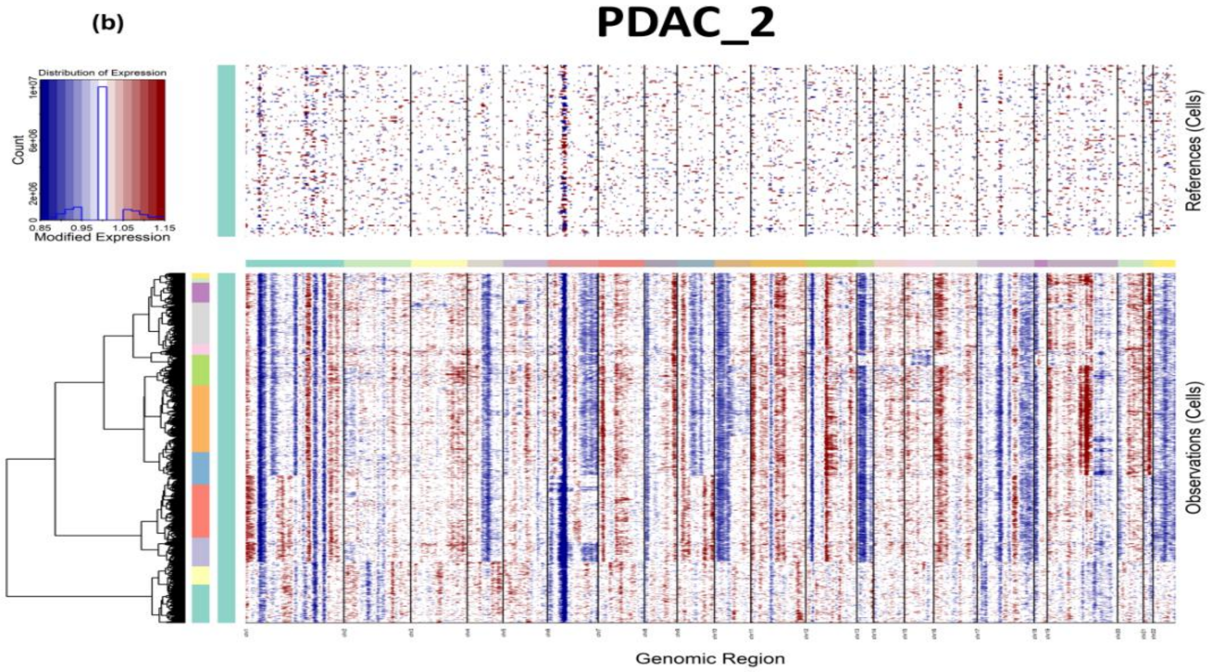

(c)

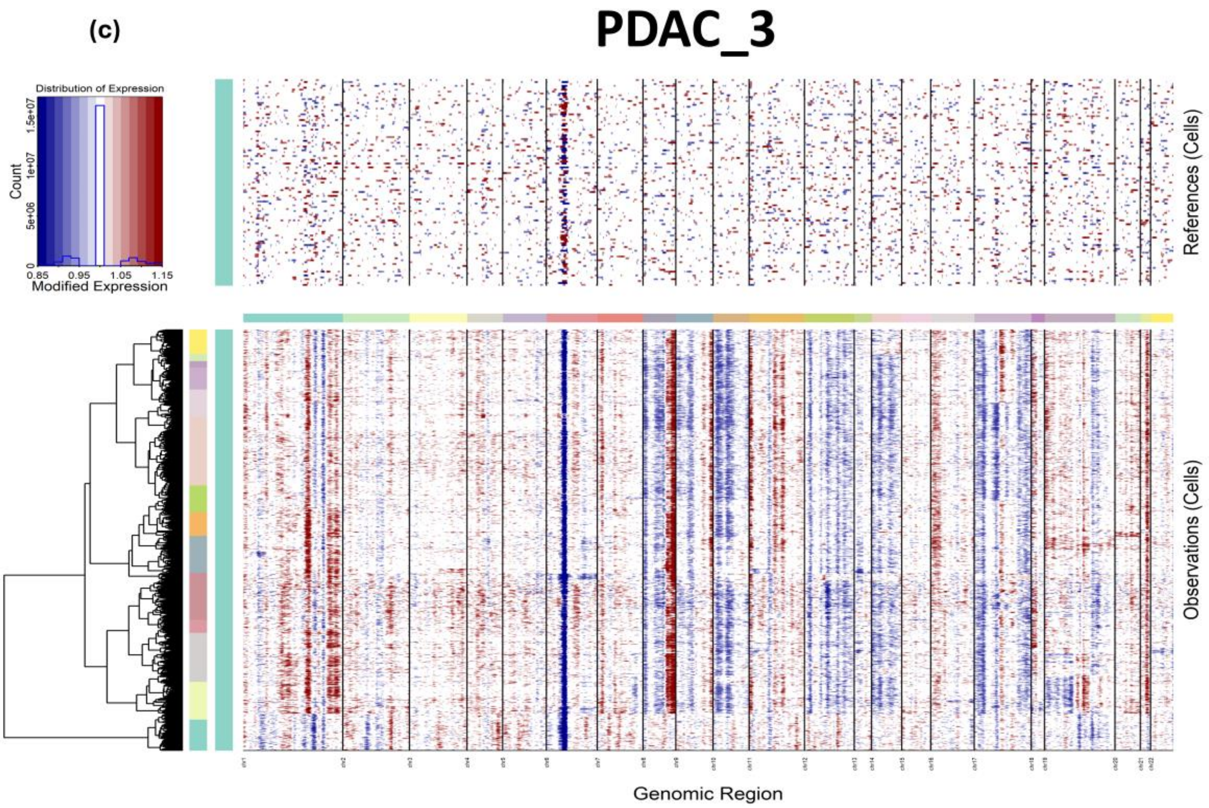
PDAC_3
Distribution of Expression
Count
Modified Expression
References (Cells)
Observations (Cells)
Genomic Region

(d)

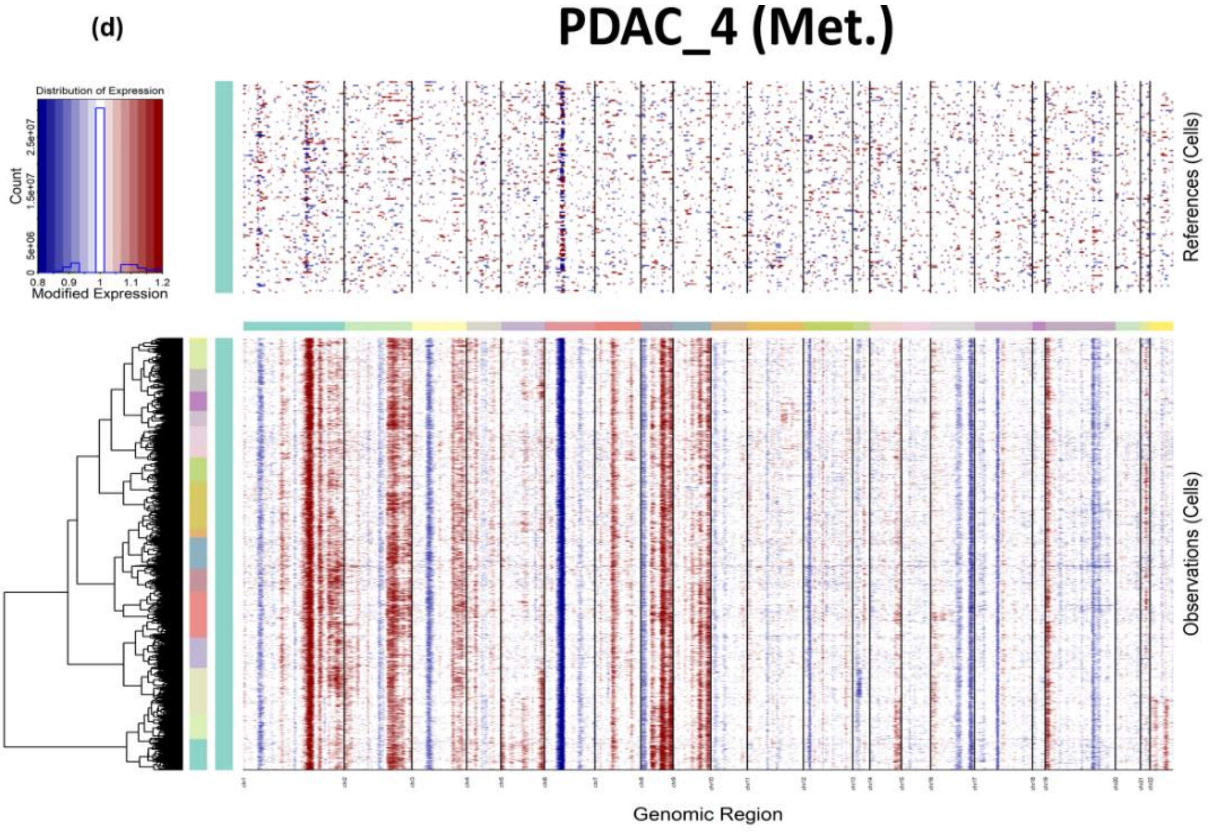
PDAC_4 (Met.)
Distribution of Expression
Count
Modified Expression
References (Cells)
Observations (Cells)
Genomic Region

(e)

PDAC_5 (Met.)

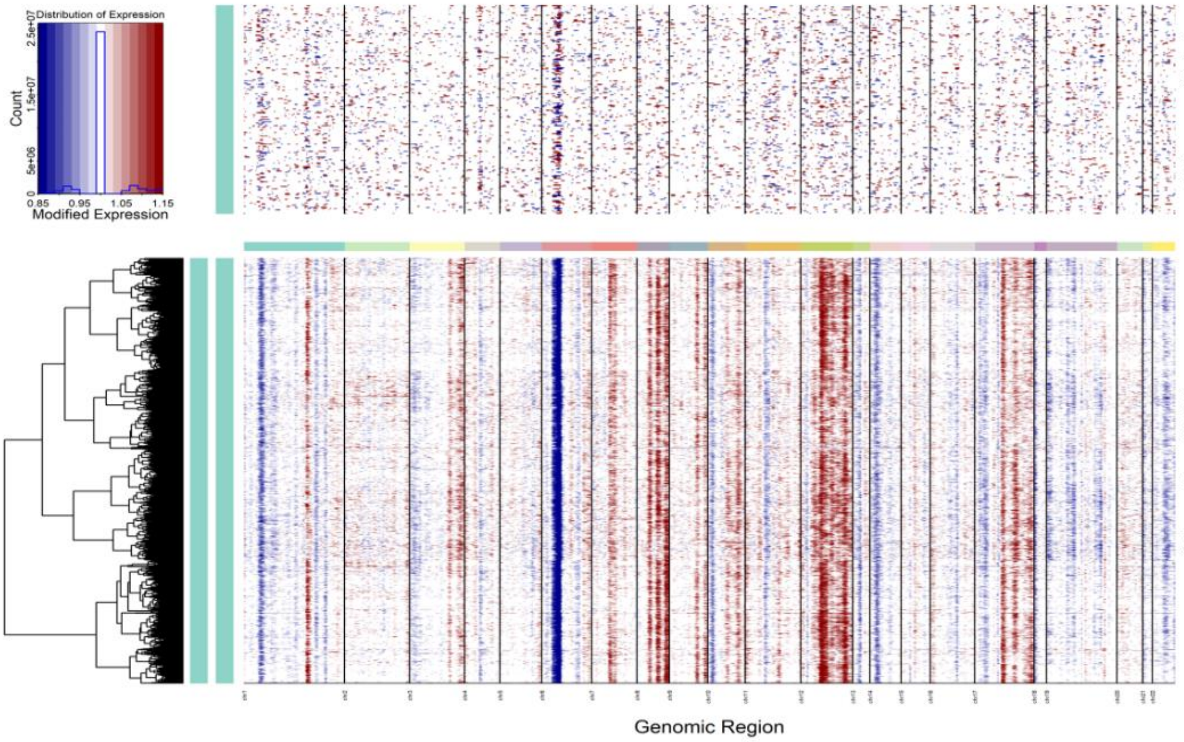

(f)

AdjNorm_1

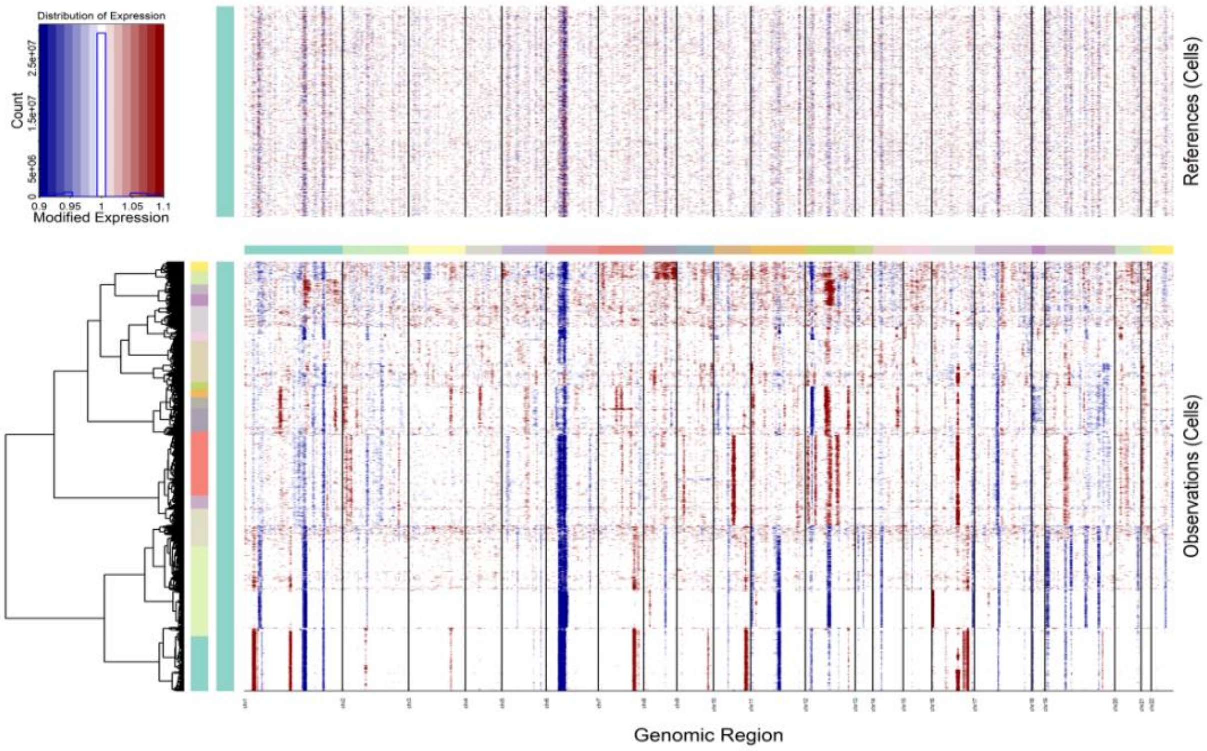

(g)

AdjNorm_2

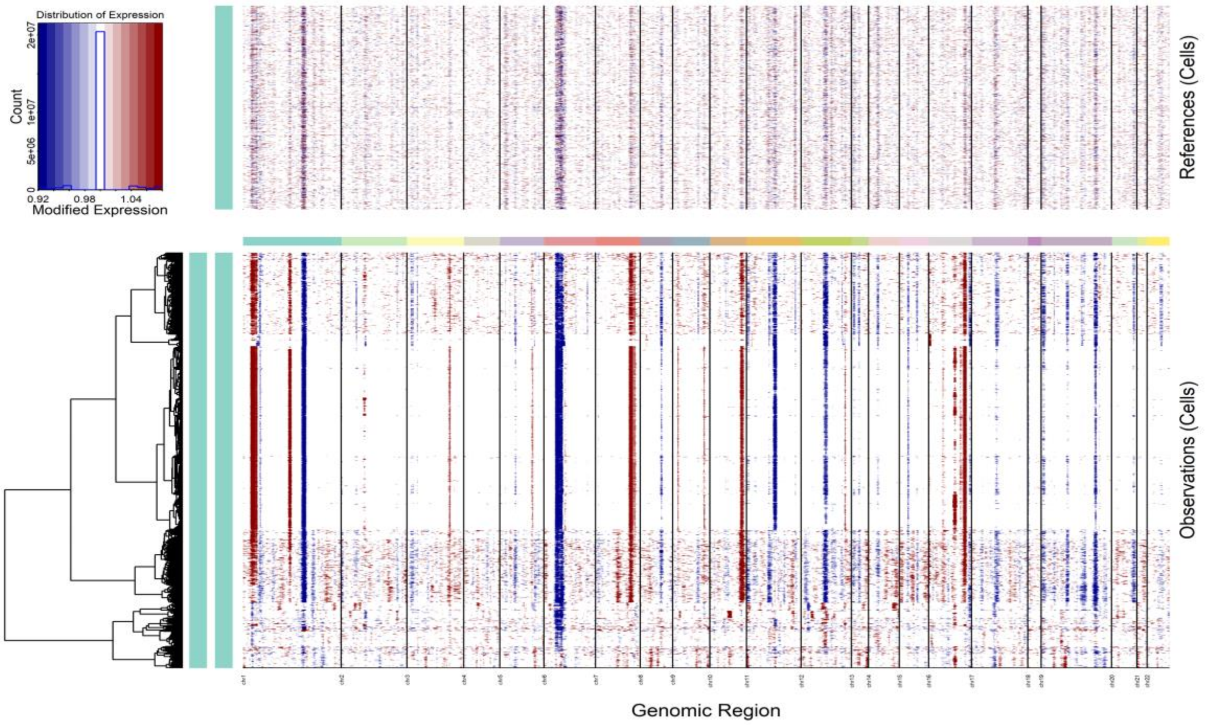

(h)

Normal_N1

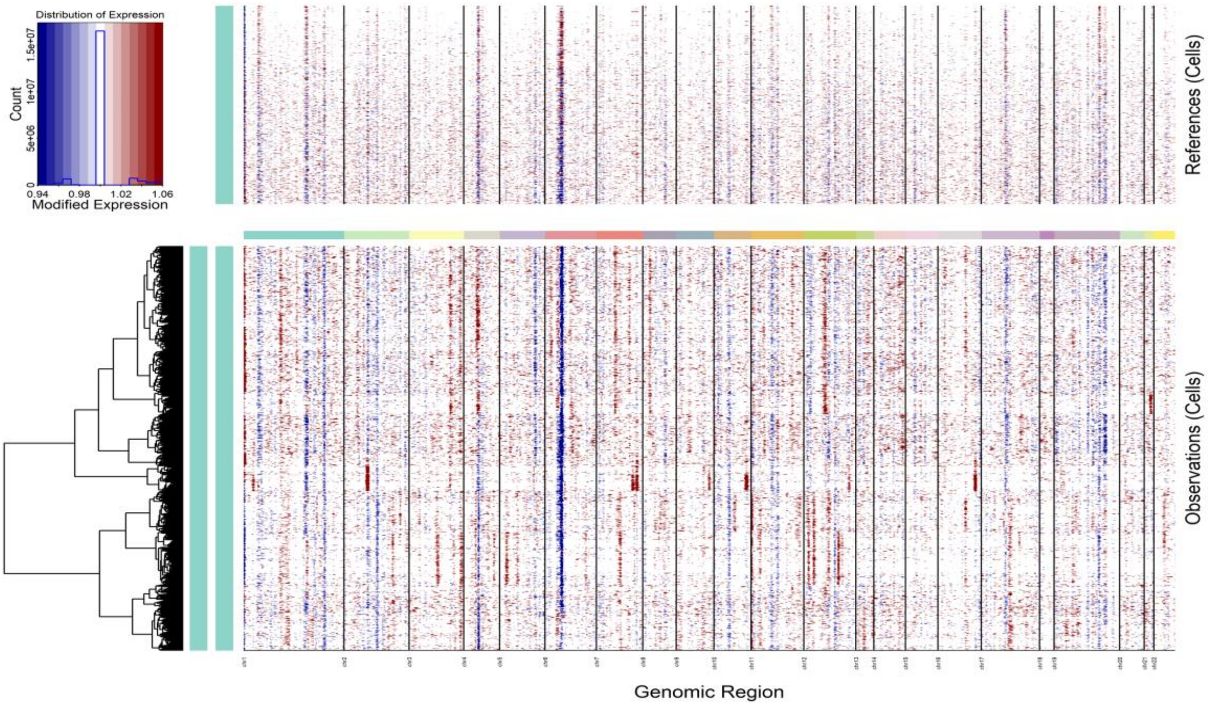

Supplementary Figure S3: CopyKAT's heatmap visualization of the CNV profiles of the primary PDAC tumor samples (**a-c**), metastatic PDAC tumor samples (**d-e**), and the control samples (**f-h**). The chromosomes are displayed on the x-axis while the distinct clusters are displayed on the y-axis. The X-chromosome is displayed as chr 23, while the Y-chromosome is not processed by tool. In comparison to normal cells, the chromosomes displayed in orange colour are likely amplified and highly expressed, whereas those with blue colour are deleted and lowly expressed. The predicted aneuploid cells had more amplification and deletion events in comparison to the predicted diploid cells.

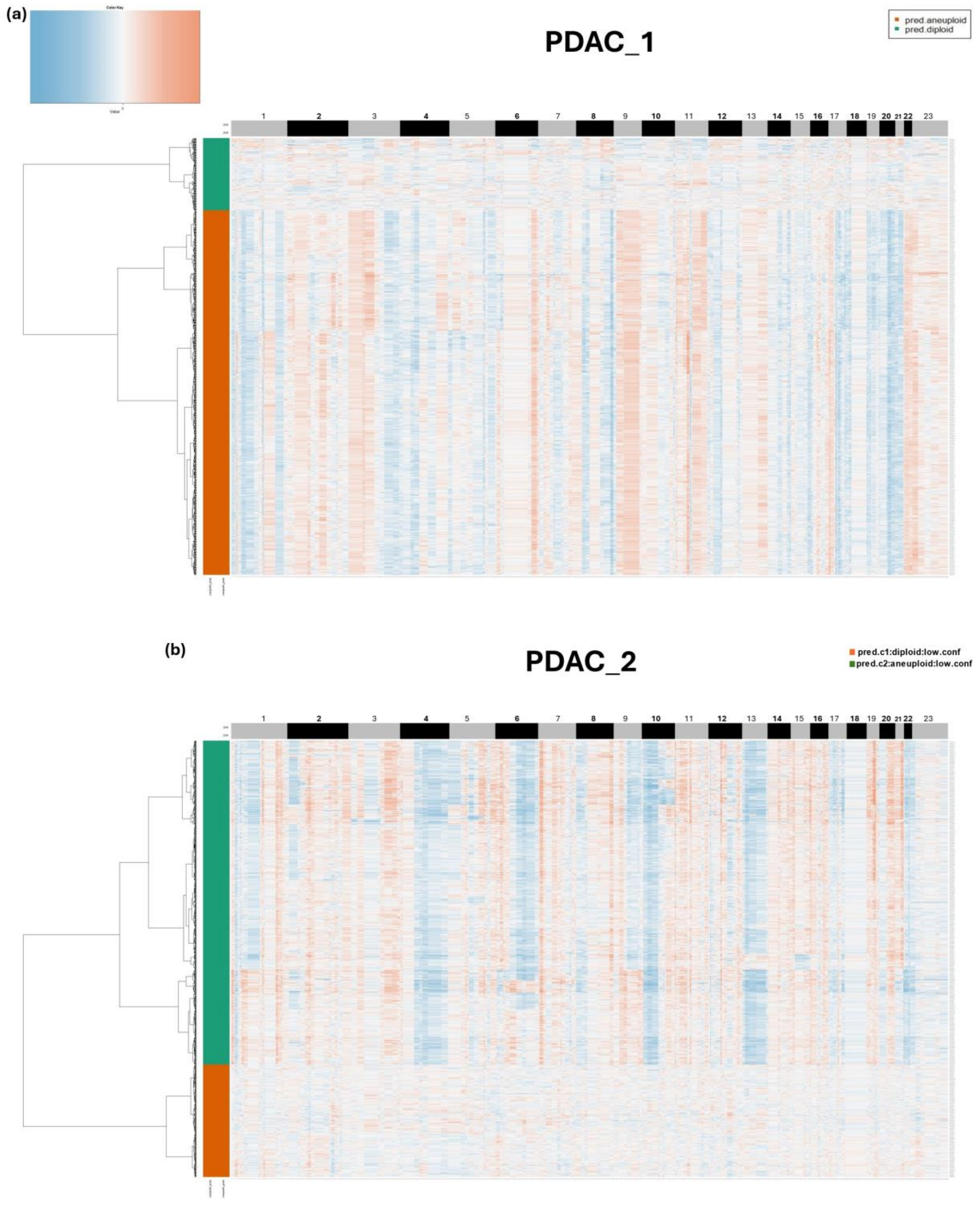

(c)

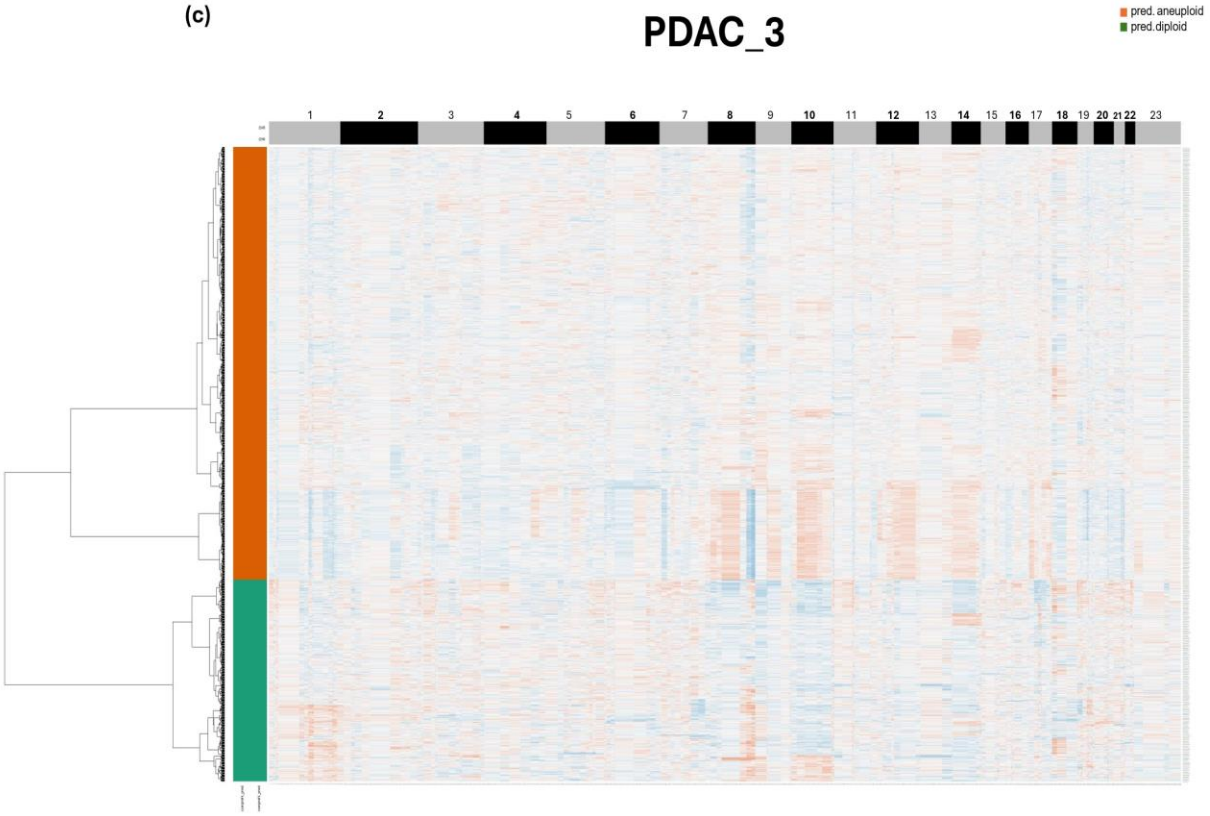
PDAC_3
pred. aneuploid
pred.diploid
1
2
3
4
5
6
7
8
9
10
11
12
13
14
15
16
17
18
19
20
21
22
23

(e)

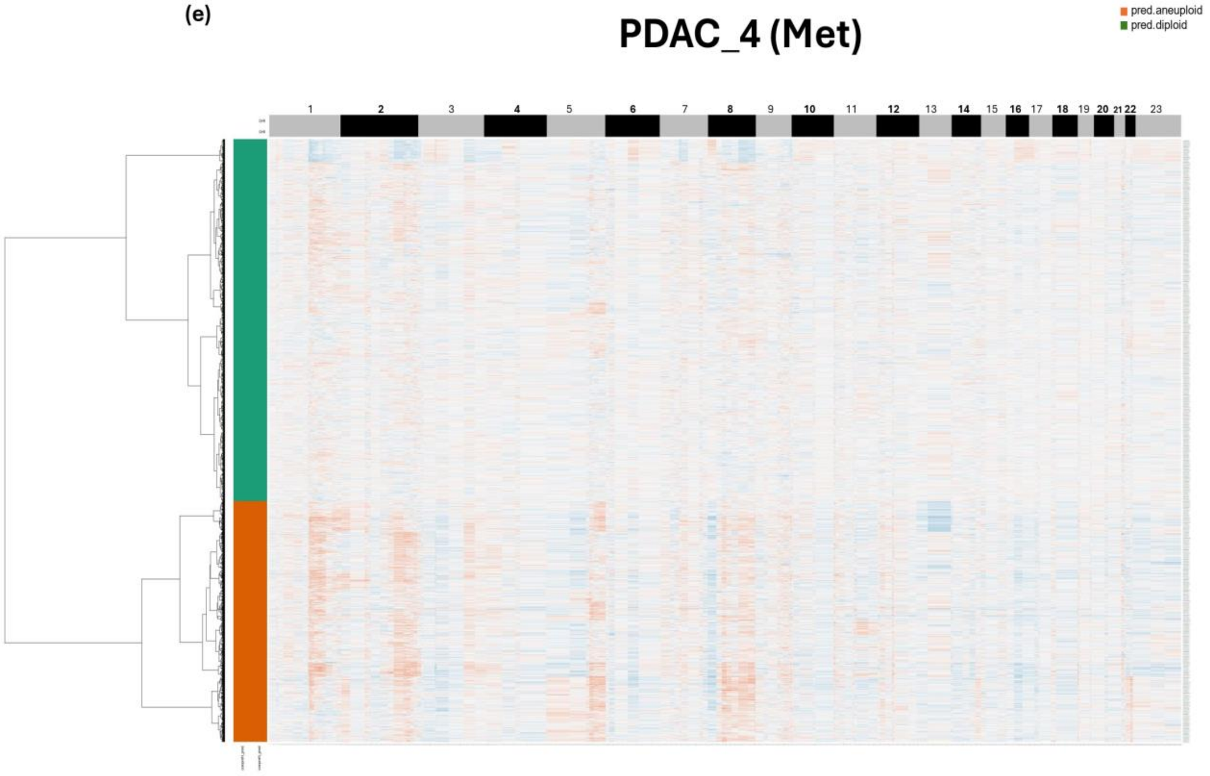
PDAC_4 (Met)
pred.aneuploid
pred.diploid
1
2
3
4
5
6
7
8
9
10
11
12
13
14
15
16
17
18
19
20
21
22
23

(d)

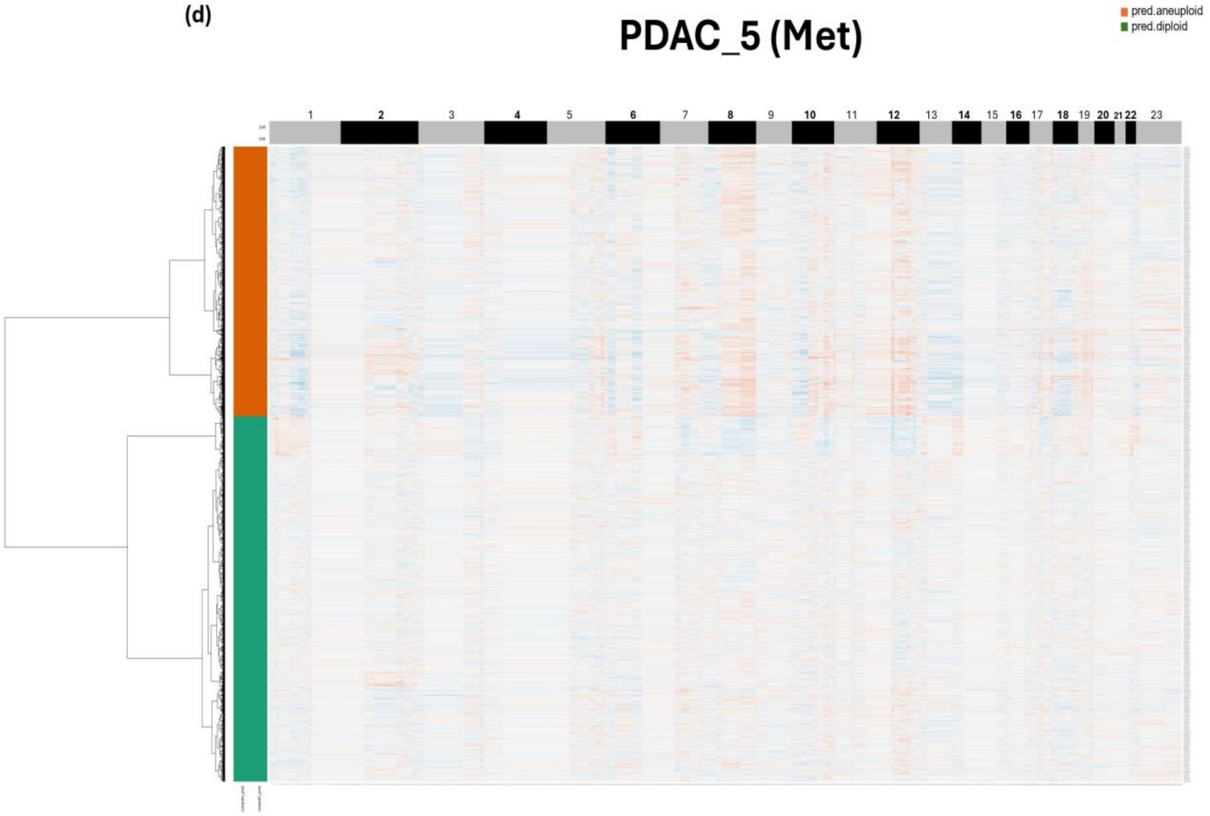
PDAC_5 (Met)
pred.aneuploid
pred.diploid
1
2
3
4
5
6
7
8
9
10
11
12
13
14
15
16
17
18
19
20
21
22
23

(f)

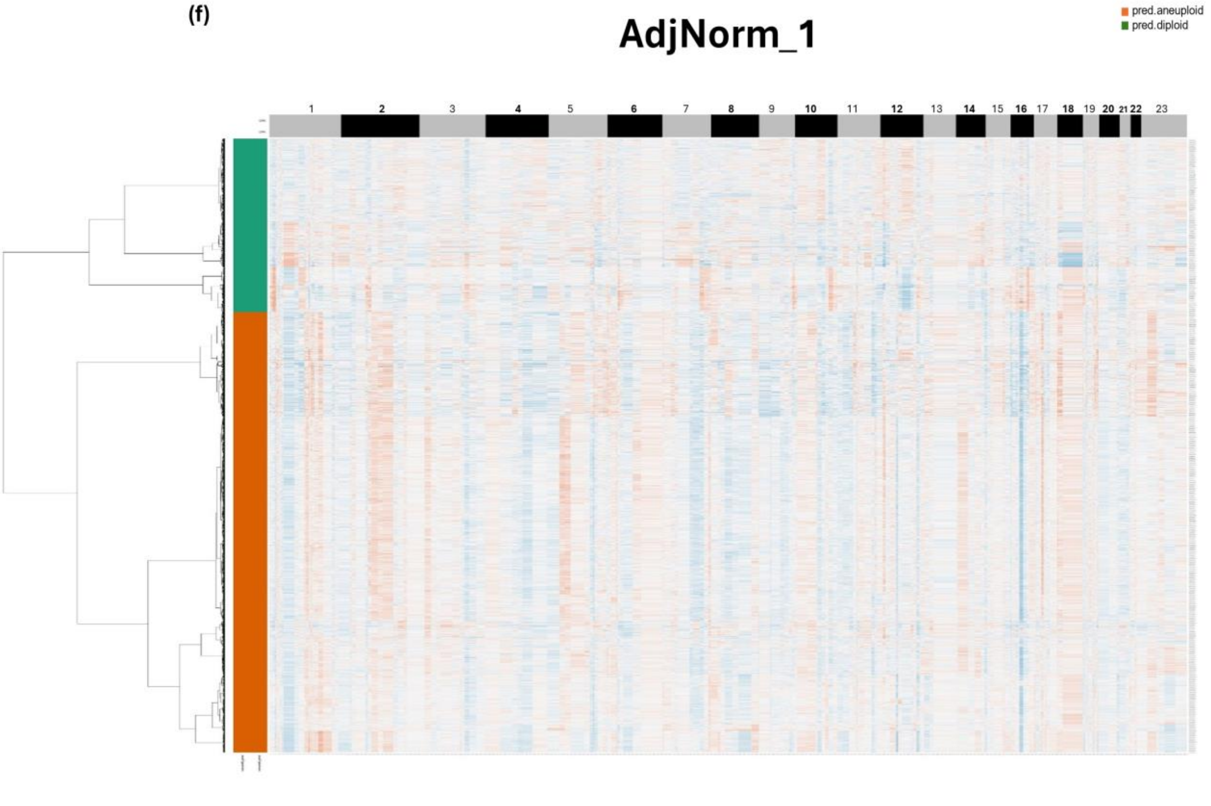
AdjNorm_1
pred.aneuploid
pred.diploid
1
2
3
4
5
6
7
8
9
10
11
12
13
14
15
16
17
18
19
20
21
22
23

(g)

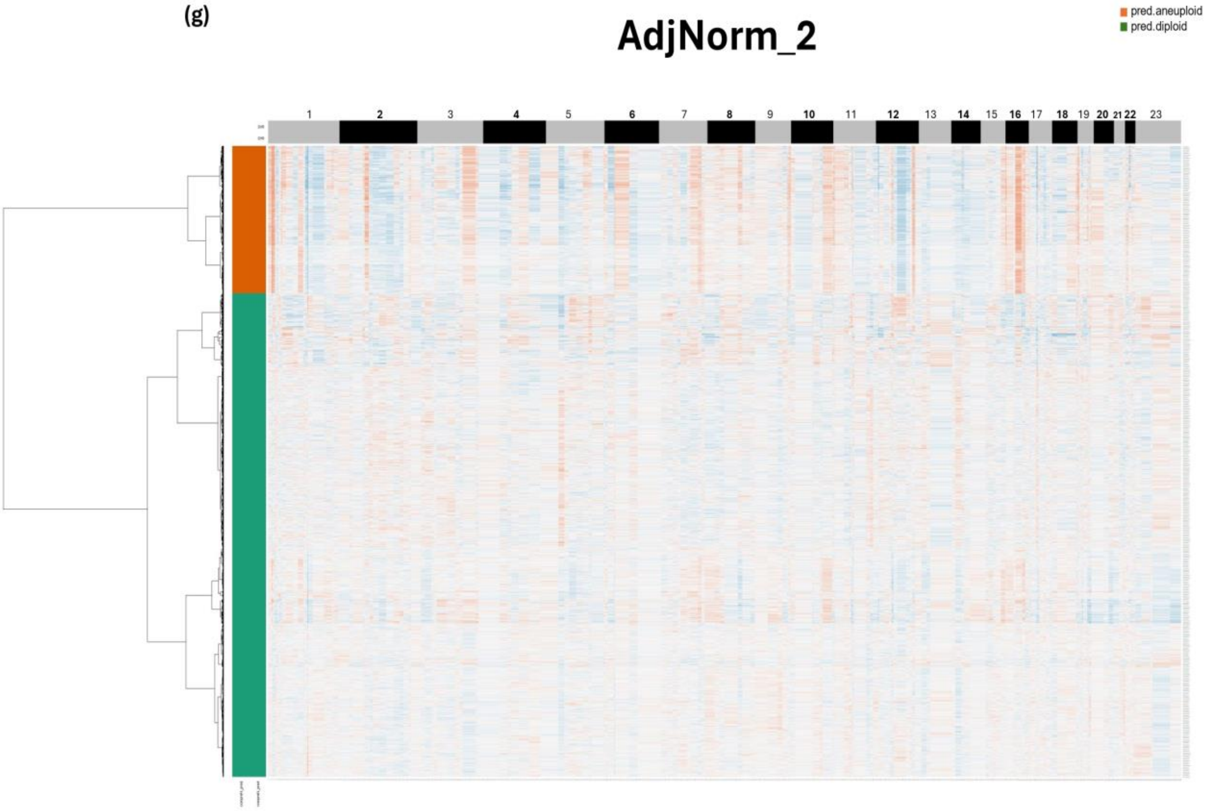
AdjNorm_2
pred.aneuploid
pred.diploid
1
2
3
4
5
6
7
8
9
10
11
12
13
14
15
16
17
18
19
20
21
22
23

(h)

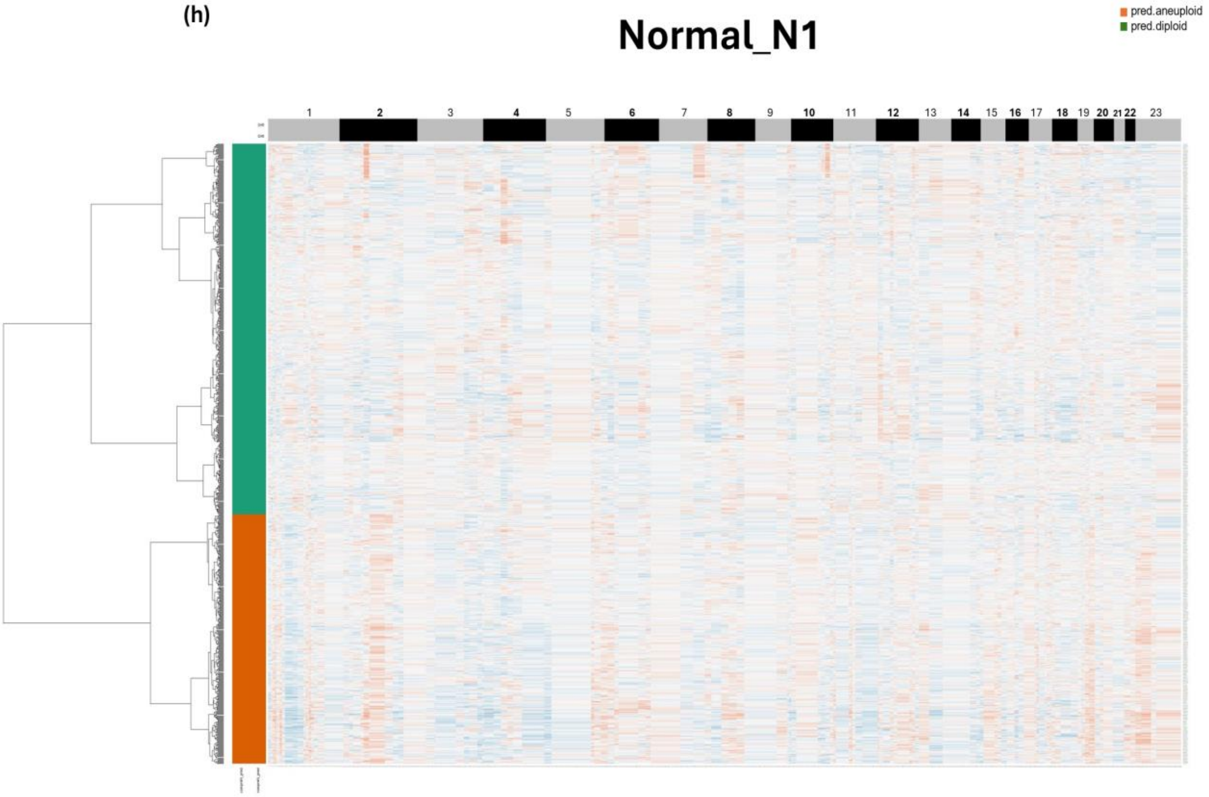
Normal_N1
pred.aneuploid
pred.diploid
1
2
3
4
5
6
7
8
9
10
11
12
13
14
15
16
17
18
19
20
21
22
23

Supplementary Figure S4: SCEVAN's heatmap visualization of the predictions and subclones of the predicted tumor cells in the primary PDAC (**a-c**), metastatic PDAC (**d-e**), and the control samples(**f-h**). For each sample, the heatmaps in the second column **(ii)** are derived from a further clusterization of the tumor cells (orange cluster) identified in the respective heatmaps on the left **(i)**. The chromosomes are indicated on the x-axis, while the distinct clusters are represented on the y-axis. The x-axis represents the individual chromosomes while the tumor status is indicated on the y-axis. The chromosomes displayed in orange colour are likely amplified and therefore highly expressed when compared to the normal cells, whereas those with blue colour are lowly expressed due to deletions. The predicted tumor cells had more deletion and amplification events in comparison to the predicted normal cells.

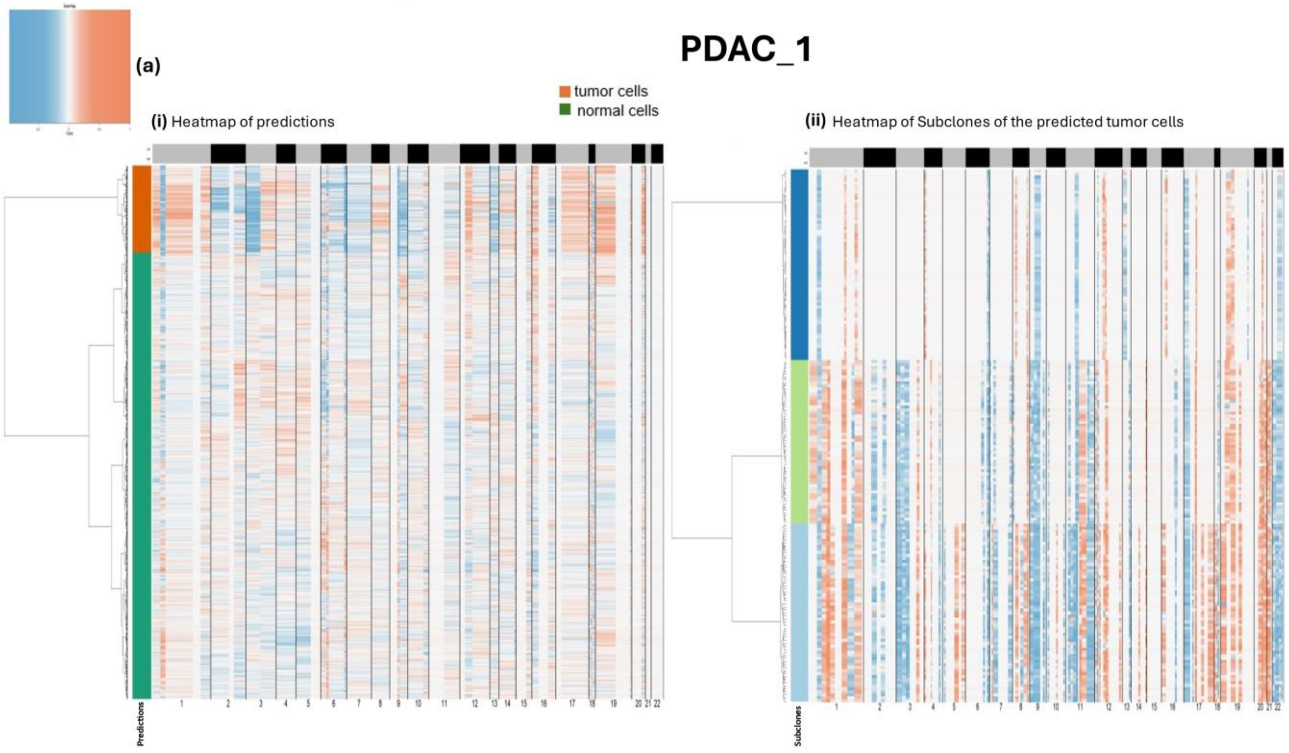

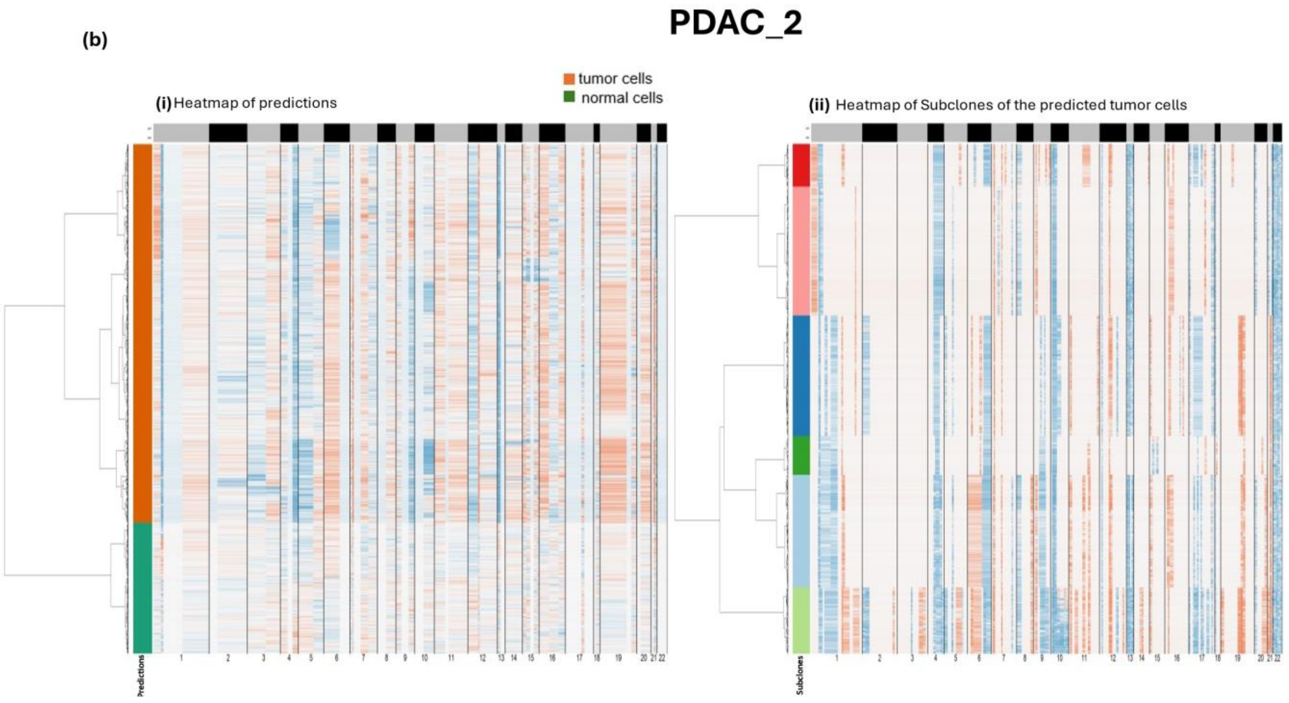

(c)

PDAC_3

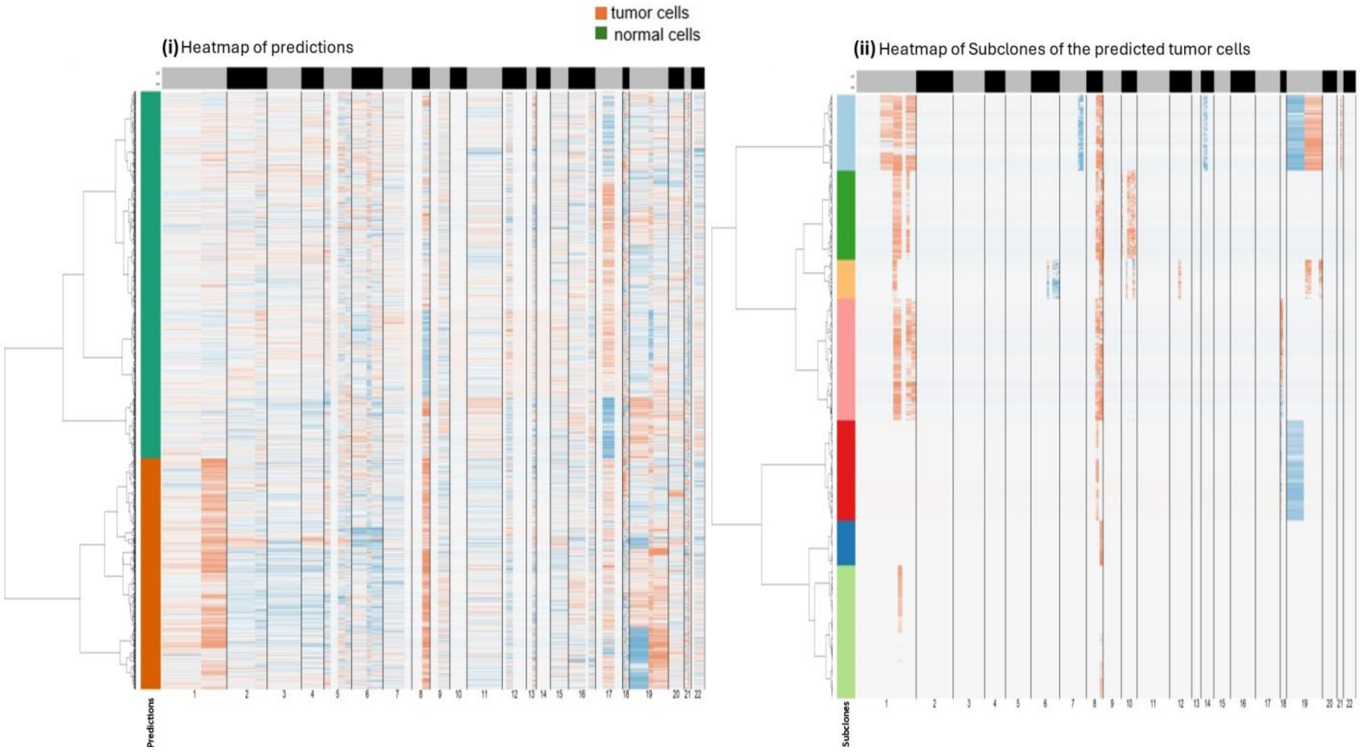

(d)

PDAC_4 (Met)

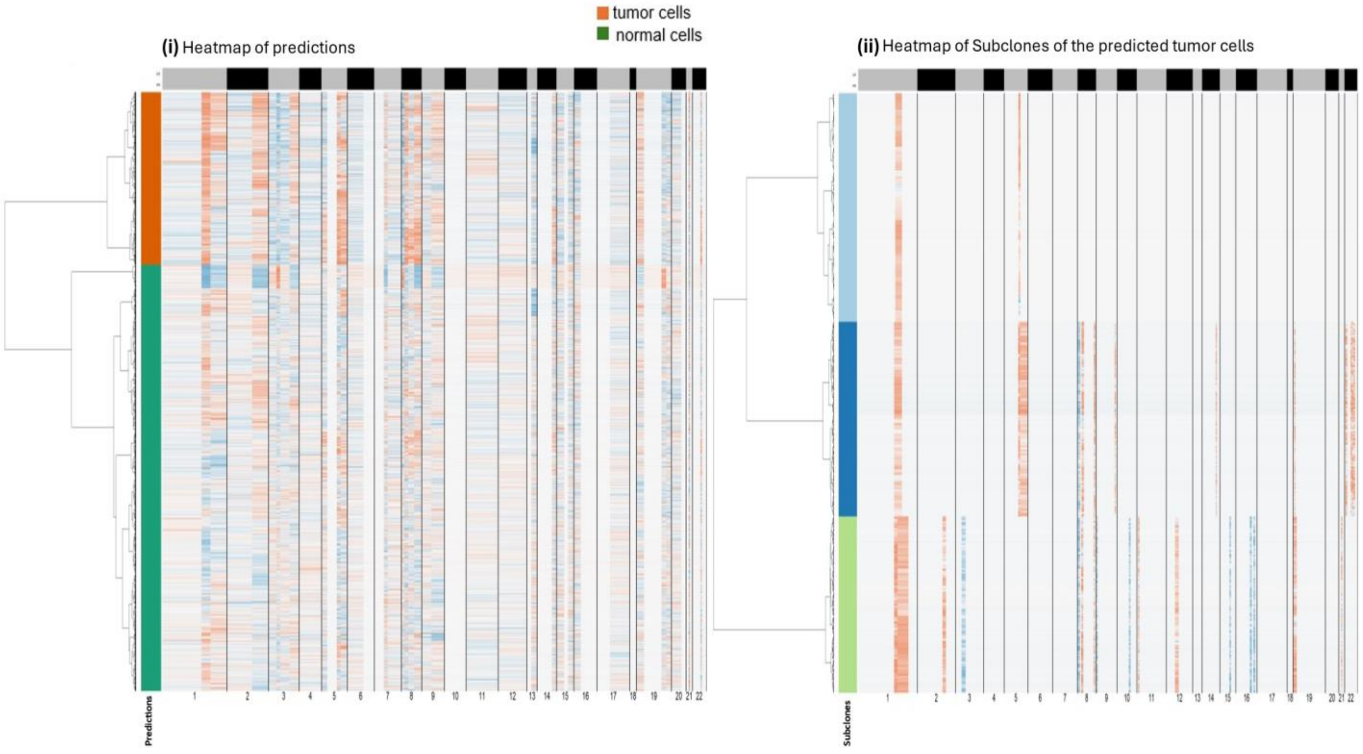

(e)

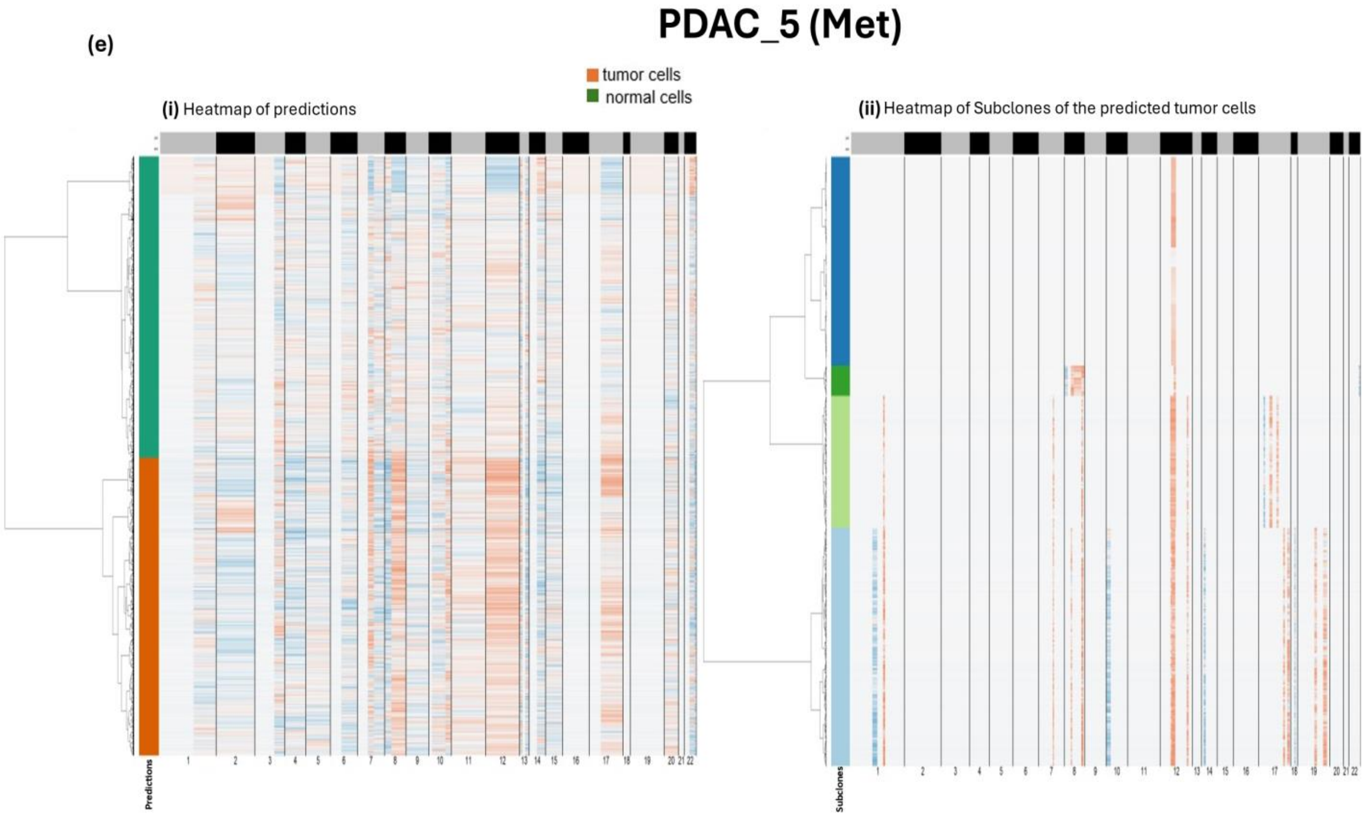
PDAC_5 (Met)
tumor cells
normal cells
(i) Heatmap of predictions
(ii) Heatmap of Subclones of the predicted tumor cells
Predictions
Subclones

(f)

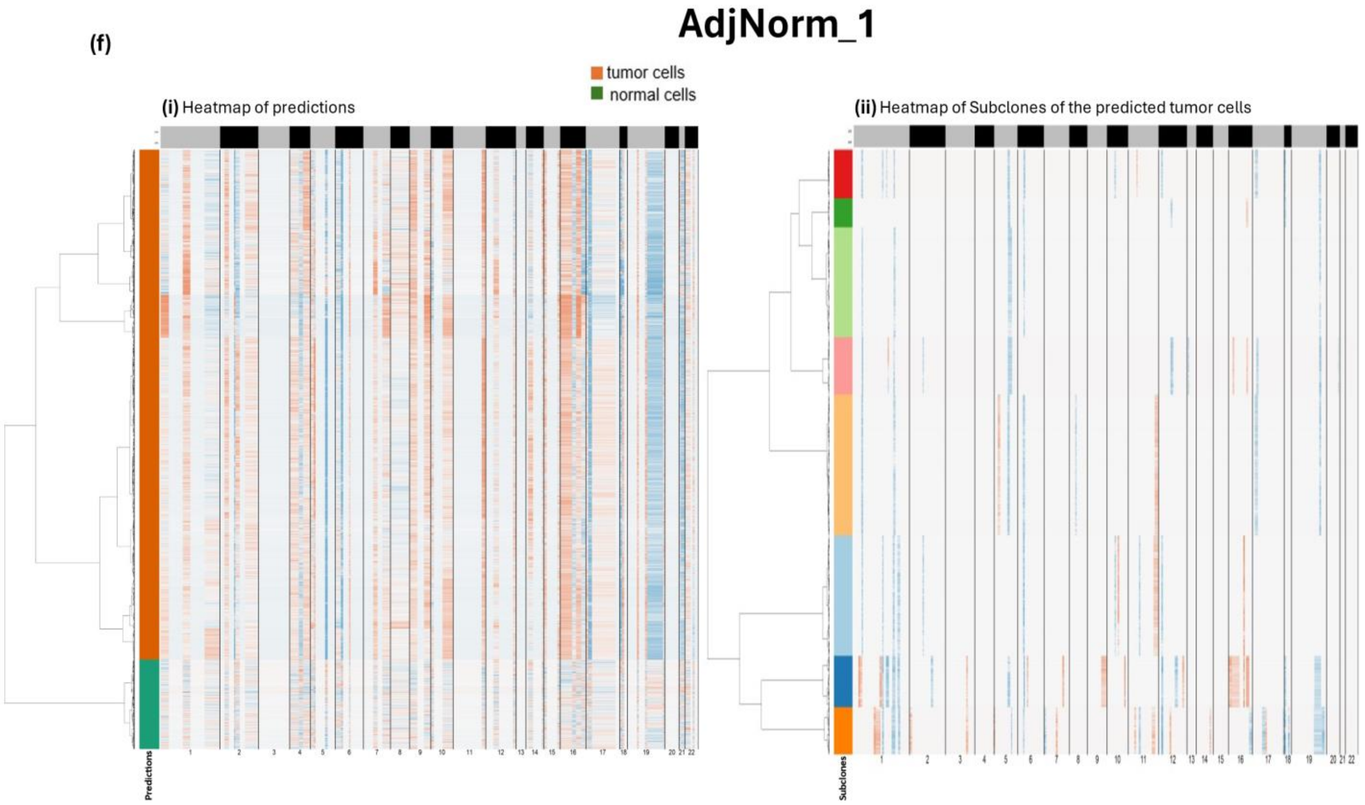
AdjNorm_1
tumor cells
normal cells
(i) Heatmap of predictions
(ii) Heatmap of Subclones of the predicted tumor cells
Predictions
Subclones

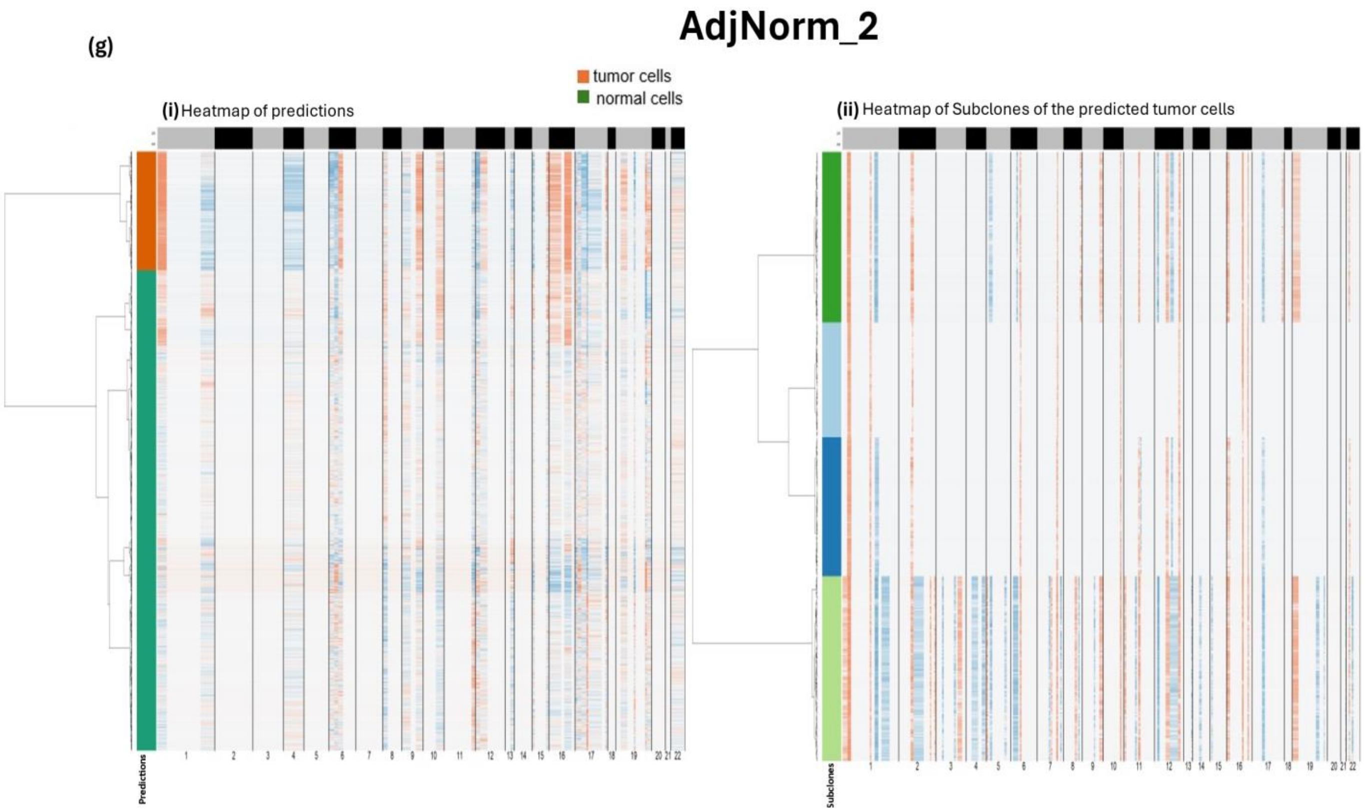
(g)
AdjNorm_2
tumor cells
normal cells
(i) Heatmap of predictions
(ii) Heatmap of Subclones of the predicted tumor cells
Predictions
Subclones

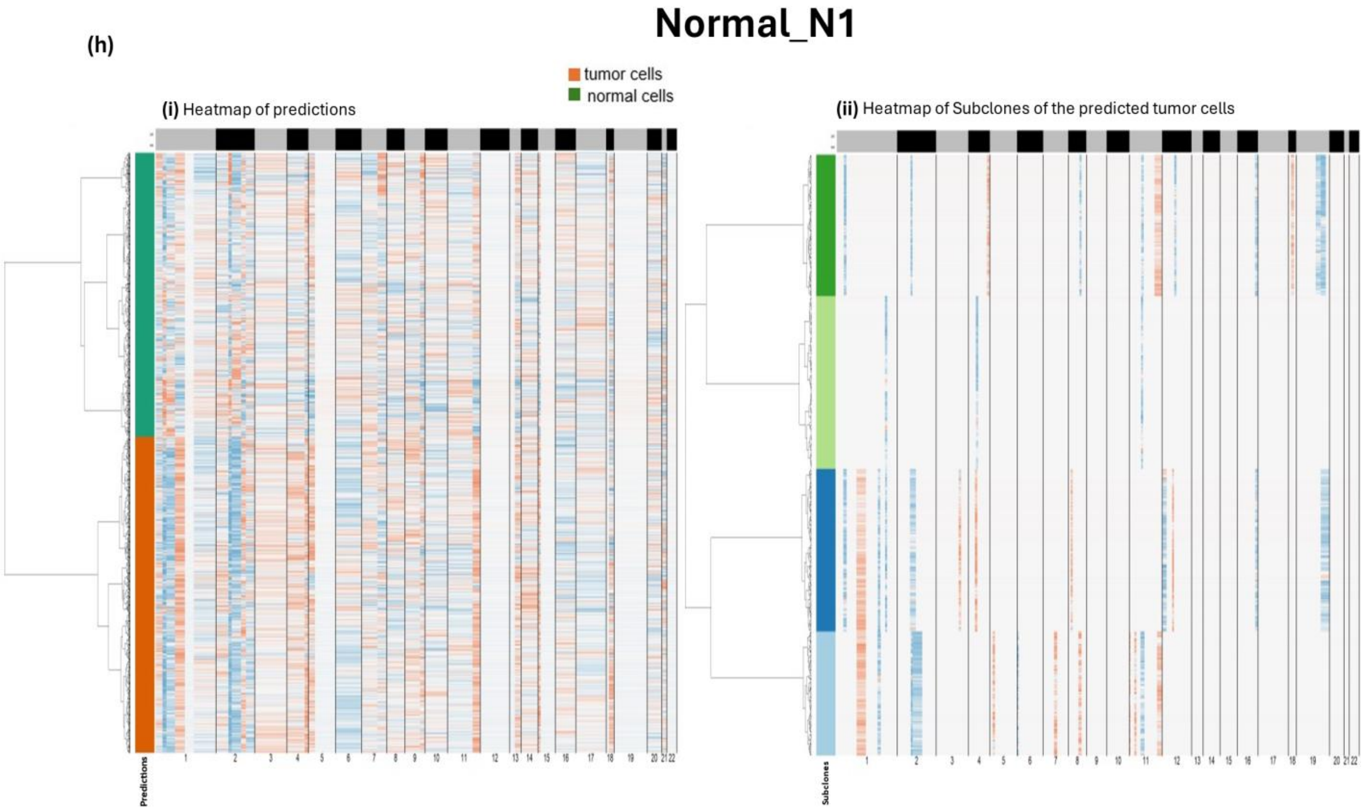
(h)
Normal_N1
tumor cells
normal cells
(i) Heatmap of predictions
(ii) Heatmap of Subclones of the predicted tumor cells
Predictions
Subclones

Supplementary Table S2: Detailed cell type annotation for each sample using SingleR.

CELL TYPE	PDAC_1	PDAC_2	PDAC_3	PDAC_4 (Met.)	PDAC_5 (Met.)	AdjNorm_1	AdjNorm_2	Normal_N1
**NON-IMMUNE CELLS**	*(34.8%)*	*(90.0%)*	*(93.2%)*	*(95.6%)*	*(93.0%)*	*(43.5%)*	*(43.8%)*	*(57.4%)*
Epithelial_cells	196	844	1325	2774	2303	705	653	388
Endothelial_cells	10	12		1	1	156	111	170
Fibroblasts	95	83	84		1	31	8	84
Tissue_stem_cells	23	27	9			529	62	127
Smooth_muscle_cells	2	20	2			23	9	83
Chondrocytes	33	26	41			23	13	31
Common Myeloid Progenitor (CMP) cells	1	3				127	73	
Erythroblast	3					90	25	
Granulocyte/Monocyte Progenitor (GMP) cells	1		2			16	27	2
Neurons	1	8				36	18	7
Pre-B_cell_CD34-					5	27	28	1
Embryonic_stem_cells						8	11	4
Bone Marrow & Progenitor (BM & Prog.) cells						2	6	
Gametocytes						3	2	1
Hepatocytes				1	1	205	370	125
Mesenchymal stem cells (MSCs)		1				1		
induced Pluripotent Stem (iPS) cells				2		30	64	7
Bone Marrow (BM) Cells	16					87	14	
Keratinocytes						3	1	
Osteoblasts						2	1	6
Megakaryocyte/Erythroid Progenitor (MEP) cells						9	12	
Platelets						9	14	
HSC_CD34+ (CD34 positive-Hematopoietic stem cells)						24	47	29
Myelocyte						8	1	
Neuroepithelial_cell	1	1				6	3	
Astrocyte						7	15	
HSC_-G-CSF (Granulocyte colony-stimulating factor positive-Hematopoietic stem cells)						12	6	
Pro-B_cell_CD34+						9	20	
Pro-Myelocyte						11	2	
**IMMUNE CELLS**	*(65.2%)*	*(10.0%)*	*(6.8%)*	*(4.4%)*	*(7.0%)*	*(55.7%)*	*(55.0%)*	*(42.6%)*
Macrophage	398	53	51	57	43	638	269	520
Neutrophils						532	59	16
Natural killer (NK) cells	6	1	9	28	19	251	183	23
Monocyte	192	28	34	31	51	256	632	88
B_cell/lymphocyte	8	3	1	1	1	88	38	
Dendritic cells (DCs)	23	26	11	7	1	53	70	118
T_cells/lyphocytes	89	3	1	3	58	952	723	25
**Total Number of Cells**	**1098**	**1139**	**1570**	**2905**	**2484**	**4969**	**3590**	**1855**

Supplementary Tables S3-S5: Detailed calculations of the performance of InferCNV **(Supplementary Table S3)**, CopyKAT **(Supplementary Table S4)** and SCEVAN **(Supplementary Table S5)** using the cells identified by markers as reference tumor cells. For each sample: True positives **(TP)** represent the overlap between the tumor cells predicted by each tool and the cells identified by markers, False Positives **(FP)** are those predicted by the tools but not by markers, False negatives **(FN)** are cells not predicted as tumorous by each tool among those identified as cancer cells by markers, and true negatives **(TN)** are those not predicted as tumorous by both markers and the CNV inference tools.

Supplementary Table S3: InferCNV

Sample	Reference tumor cell number	InferCNV					
		TP	FP	TN	FN	Sensitivity	Specificity
**PDAC_1**	146	145	834	118	1	0.99	0.12
**PDAC_2**	737	737	295	107	0	1	0.27
**PDAC_3**	1184	1180	290	96	4	1	0.25
**PDAC_4 (Met)**	2642	2640	159	104	2	1	0.4
**PDAC_5 (Met)**	2191	1881	86	207	310	0.86	0.71
**AdjNorm_1**	271	43	962	3736	228	0.16	0.8
**AdjNorm_2**	50	3	34	3506	47	0.06	0.99
**Normal_N1**	0		0	1855	0		1
						**Mean**	
						0.72	*0.57*

Supplementary Table S4: CopyKAT

Sample	Reference tumor cell number	CopyKAT					
		TP	FP	TN	FN	Sensitivity	Specificity
**PDAC_1**	146	15	884	68	131	0.1	0.07
**PDAC_2**	737	734	110	292	3	1	0.73
**PDAC_3**	1184	719	347	39	465	0.61	0.1
**PDAC_4 (Met)**	2642	1044	114	149	1598	0.4	0.57
**PDAC_5 (Met)**	2191	952	67	226	1239	0.43	0.77
**AdjNorm_1**	271	32	2249	2449	239	0.12	0.52
**AdjNorm_2**	50	3	450	3090	47	0.06	0.87
**Normal_N1**	0	0	359	1496	0		0.81
						**Mean**	
						0.39	*0.55*

Supplementary Table S5: SCEVAN

Sample	Reference tumor cell number	SCEVAN					
		TP	FP	TN	FN	Sensitivity	Specificity
**PDAC_1**	146	125	51	901	21	0.86	0.95
**PDAC_2**	737	733	113	289	4	0.99	0.72
**PDAC_3**	1184	540	64	322	644	0.46	0.83
**PDAC_4 (Met)**	2642	731	105	158	1911	0.28	0.6
**PDAC_5 (Met)**	2191	811	56	237	1380	0.37	0.81
**AdjNorm_1**	271	246	2572	2126	25	0.91	0.45
**AdjNorm_2**	50	3	422	3118	47	0.06	0.88
**Normal_N1**	0	0	518	1337	0		0.72
						**Mean**	
						0.56	*0.75*

Supplementary Table S6: This table shows how variations in parameter settings affect the results in some samples. In one row of the table, we show the number of tumor cells predicted by the tool as a function of the parameter settings. In the next row we show the number of true tumor cells present among those predicted to be tumorous by the tools. It can be observed that changing these parameter settings has no effect in one sample (PDAC_2) while the effects are significant and non-linear in another sample (PDAC_4). In other words, the true tumor cells in the predictions do not progressively increase or decrease as the parameter varies. Hence one can realize that the prediction results strongly depend on the sample as well as on the parameters.

**SAMPLES**		**SCEVAN**							**CopyKAT**				
		**beta_vega values**							**Ks.Cut values**				
		**0.05**	**0.1**	**0.5 (default)**	**1.0**	**1.5 (used)**	**2.0**	**2.5**	**0.05**	**0.1 (default and used)**	**0.15**	**0.2**	**0.25**
**PDAC_2** (737 true cancer cells)	Predicted cancer cells	845	828	844	846	846	845	848	844	844	847	844	589
	True tumor cells among those predicted	734	732	734	734	733	734	734	734	734	734	734	496
**PDAC_4 (Met)** (2642 true cancer cells)	Predicted cancer cells	1135	981	846	1448	836	1023	1236	995	1158	1350	1057	1226
	True tumor cells among those predicted	1017	873	738	1327	731	910	1119	893	1044	1240	955	1116
